# Modelled estimates of hospitalisations attributable to respiratory syncytial virus and influenza in Australia, 2009–2017

**DOI:** 10.1111/irv.13003

**Published:** 2022-06-30

**Authors:** Allen L. Nazareno, David J. Muscatello, Robin M. Turner, James G. Wood, Hannah C. Moore, Anthony T. Newall

**Affiliations:** ^1^ School of Population Health, Faculty of Medicine University of New South Wales Sydney New South Wales Australia; ^2^ Institute of Mathematical Sciences and Physics, College of Arts and Sciences University of the Philippines Los Baños Laguna Philippines; ^3^ Biostatistics Centre, Division of Health Sciences University of Otago Dunedin New Zealand; ^4^ Wesfarmers Centre of Vaccines and Infectious Diseases, Telethon Kids Institute University of Western Australia Perth Western Australia Australia

**Keywords:** burden, epidemiology, influenza hospitalisation, regression modelling, RSV hospitalisation, time series analysis

## Abstract

**Background:**

Respiratory syncytial virus (RSV) and influenza are important causes of disease in children and adults. In Australia, information on the burden of RSV in adults is particularly limited.

**Methods:**

We used time series analysis to estimate respiratory, acute respiratory infection, pneumonia and influenza, and bronchiolitis hospitalisations attributable to RSV and influenza in Australia during 2009 through 2017. RSV and influenza‐coded hospitalisations in <5‐year‐olds were used as proxies for relative weekly viral activity.

**Results:**

From 2009 to 2017, the estimated all‐age average annual rates of respiratory hospitalisations attributable to RSV and seasonal influenza (excluding 2009) were 54.8 (95% confidence interval [CI]: 20.1, 88.8) and 87.8 (95% CI: 74.5, 97.7) per 100,000, respectively. The highest estimated average annual RSV‐attributable respiratory hospitalisation rate per 100,000 was 464.2 (95% CI: 285.9, 641.2) in <5‐year‐olds. For seasonal influenza, it was 521.6 (95% CI: 420.9, 600.0) in persons aged ≥75 years. In ≥75‐year‐olds, modelled estimates were approximately eight and two times the coded estimates for RSV and seasonal influenza, respectively.

**Conclusions:**

RSV and influenza are major causes of hospitalisation in young children and older adults in Australia, with morbidity underestimated by hospital diagnosis codes.

## 1 INTRODUCTION

Respiratory syncytial virus (RSV) and influenza virus are important causes of infectious respiratory illness and result in substantial severe disease burden, including hospitalisation and death.^1^, ^2^, ^3^ In 2015, RSV was estimated to cause around 33 million acute lower respiratory infections, 3.2 million hospitalisations, and about 60,000 in‐hospital deaths in young children globally.^3^ RSV has also been recognised to pose a considerable risk of hospitalisation and death in older adults.^2^, ^4^ The World Health Organization (WHO) estimates 1 billion influenza cases currently occur annually worldwide.^5^ Seasonal influenza has been responsible for substantial morbidity and mortality in children and an even greater burden in older adults.^1^, ^6^

The reliance on pathogen‐specific diagnostic codes in healthcare databases can lead to underestimation of the disease burden from infectious diseases^7^ due to the inability to capture cases where the pathogen has not been laboratory confirmed. Time series regression modelling has frequently been used to estimate the population disease burden from seasonal infectious diseases^8^, ^9^, ^10^, ^11^, ^12^, ^13^, ^14^, ^15^, ^16^, ^17^, ^18^, ^19^, ^20^, ^21^, ^22^, ^23^, ^24^ as it can provide estimates of the total attributable disease burden, including undiagnosed cases. This approach has been previously used to assess the burden of influenza,^8^, ^9^, ^10^, ^11^, ^12^, ^13^, ^14^, ^15^, ^16^, ^17^, ^18^, ^19^, ^20^, ^21^ and the application of these methods to estimate RSV‐attributable burden has grown in the past decade.^16^, ^17^, ^18^, ^19^, ^20^, ^21^, ^22^, ^23^, ^24^ However, to date, these methods have not been applied to estimate RSV‐attributable hospitalisations in Australia.

These statistical models relate changes in an indicator of the relative activity of a specific infection in the population to changes in the relative incidence of a non‐specific health outcome category (e.g., all respiratory illness)^1^, ^18^, ^19^, ^21^, ^23^ to estimate the proportion of that health outcome attributable to the virus. Typically, time series of counts or rates of laboratory‐confirmed infections are used as indicators for the measure of virus activity. However, unlike influenza, RSV was not notifiable to health authorities in Australia during the study period.^25^ In the absence of such data, time series of counts or rates of diagnostic codes recorded in the health outcome data specific to an identified virus (e.g., hospitalisations coded as being due to RSV or influenza) can be used as a proxy indicator of relative virus activity.^14^, ^21^

While there is no licensed RSV vaccine currently available, several candidates are in different phases of clinical trials.^26^ These candidate vaccines target various population groups, including older adults.^27^ This highlights the need to better quantify the burden of RSV for all age groups to evaluate the benefits of these potential vaccination strategies. Estimates of influenza burden are also important to inform influenza mitigation efforts and to differentiate the burden of these important respiratory viruses.

We estimated age‐specific hospitalisation attributable to RSV and influenza in Australia annually and overall for 2009 through 2017, using multiple regression modelling of time series data obtained from a national hospitalisation outcome database.

## 2 METHODS

### 2.1 Data sources and preparation

From the National Hospital Morbidity Database (NHMD),^28^ we obtained non‐identified records of patients who were admitted and discharged (‘separated’) from hospital during the study period, 1 July 2008 through 30 June 2018, that had International Classification of Diseases 10th Revision—Australian modification (ICD‐10‐AM) principal diagnoses of J00–J99 (‘diseases of the respiratory system’). The NHMD is a comprehensive collection of records for all episodes of care from patients admitted in almost all public and private hospitals in Australia.^28^, ^29^

Each admission record included a principal diagnosis and up to nine additional diagnoses (secondary). All diagnoses were coded according to the ICD‐10‐AM edition for each year.^30^ The individual record also contained other information, including the age group of a person at the time of admission, the urgency of admission and the length of stay.

We excluded records without an admission date (week), which was not provided when the length of stay was more than 30 days. We also excluded elective admissions (non‐emergency).^31^ One week was removed from the end of the time series as some admissions were not yet separated from the hospital, and this caused an artefactual drop at the end of the time series.

We considered the following hospital outcome categories, used in previous studies,^20^, ^21^, ^23^, ^24^ in the analysis based on the ICD‐10‐AM codes: bronchiolitis (ICD J21), pneumonia and influenza (P&I; ICD J09–J18), acute respiratory infections (ARI; ICD J00–J22 and J44.0) and all respiratory conditions (ICD J00–J99). Only principal diagnoses were considered to avoid multiple counting of admissions.

Individual records were aggregated to a weekly time series, by admission date (week), of each outcome of interest for the entire study period and stratified into age groups: 0–4, 5–14, 15–44, 45–64, 65–74 and ≥75 years. For bronchiolitis, we only included admissions for children aged <5 years as this is primarily a disease of infants.^32^ Lastly, age‐specific hospitalisation rates per 100,000 population were calculated using the Australian age‐specific population from the Australian Bureau of Statistics (ABS).^33^

### 2.2 Measure of RSV and influenza activity

We used weekly rates per 100,000 population of coded RSV and influenza hospital admissions among children aged <5 years as the proxy measure of the relative weekly activity of RSV and influenza infections. We included admissions with a principal or other diagnosis of identified RSV (ICD J12.1, J20.5, J21.0, B97.4) or influenza virus (ICD J09, J10.0, J10.1, J10.8) infection.^34^, ^35^ Unlike older age groups, this age group was not as affected by the increase in testing over time,^36^ making it a preferable surrogate to represent the activity of RSV and influenza infections across the population.

### 2.3 Statistical modelling

We used a multiple linear time series regression model, as applied in previous modelling studies,^17^, ^18^, ^22^, ^23^, ^24^ to estimate the hospitalisation burden attributable to RSV and influenza. The statistical approach involves regressing the hospital outcome variable against the proxy of virus activity variables, together with potential temporal confounding variables, to estimate a measure of association between hospitalisations and the pathogens.

The same regression model was applied to each diagnostic category and each age stratum. The complete model was defined as follows:

$$Y_{i,j}\left(t\right)=\beta_{0}+\beta_{1}t+\beta_{2}\left(\mathrm{RS}V_{t}\right)+\beta_{3}\left(\text{Influenz}a_{t}\right)+\beta_{4}\;\cos\left(\frac{2\mathrm{\pi t}}{52.182}\right)+\beta_{5}\;\sin\left(\frac{2\mathrm{\pi t}}{52.182}\right)+\sum_{k=6}^{15}\beta_{k}\left(\text{Holida}y_{k}\right)\;$$

where $Y_{i,j}\left(t\right)$ is the weekly rate of hospitalisation per 100,000 population for each week t, age group i and outcome j. The proxy variables $\mathrm{RS}V_{t}$ and $\text{influenz}a_{t}$ are the weekly rates of RSV‐coded and influenza‐coded hospitalisations per 100,000 in children aged <5 years, respectively. All rates were obtained using weekly age‐specific population denominators derived through linear interpolation between mid‐year resident population estimates from the ABS.^33^ The trigonometric terms $\cos\left(\frac{2\mathrm{\pi t}}{52.182}\right)$ and $\sin\left(\frac{2\mathrm{\pi t}}{52.182}\right)$, originally used by Serfling,^37^ represent the annual background (i.e., non‐RSV and non‐influenza) seasonal variation in hospital admission rates. The period of the trigonometric terms (the denominator) represents the average annual number of weeks across the study period. A linear term for week number, t, was included to account for any long‐term temporal trend in the hospitalisation rate. Holiday variables for the Christmas, New Year and Easter periods were incorporated into the model^10^ to account for the influence on infectious diseases activity and the provision of hospital services (see supporting information for details).

We obtained the age‐specific weekly hospitalisation rates attributable to each virus by the product of the virus‐associated parameter estimate (i.e., $\beta_{2}\;\text{or}\;\beta_{3}$) and the corresponding value of the proxy variable for each diagnostic category. We also obtained weekly estimates of virus‐attributable hospitalisation counts by multiplying the weekly rate estimates by the corresponding weekly population (and dividing by 100,000). The all‐age estimated RSV‐ and influenza‐attributable hospitalisations were determined by summing the corresponding age‐specific weekly hospitalisation counts. Both crude and age‐standardised virus‐attributable all‐age hospitalisation rates were obtained (see supporting information for details); however, we only reported the crude rates as the two rates were comparable (Table S9).

The weekly virus‐attributable hospitalisations were then summed over each year to calculate the annual RSV‐ and influenza‐attributable hospitalisations. To calculate the annual rates of RSV‐ and influenza‐attributable hospitalisation, the cumulative number of hospitalisations attributed to each virus was divided by the mid‐year population estimate for each year.

Annual estimates were averaged for the study period to produce the estimated average annual rate and count. While the half‐year periods in 2008 and 2018 were included in the modelling process, these partial years were excluded from calculating the annual averages (for the entire period) and not reported as individual annual estimates. For seasonal influenza only, 2009 (pandemic year) was also excluded in calculating the average annual estimates to distinguish the results for the pandemic year from the seasonal influenza period.

### 2.4 Model assessment and sensitivity analysis

We used a bootstrapping approach applied by Goldstein *et al* ^8^ to obtain confidence intervals (CIs) for the model parameters in the presence of autocorrelation in the residuals (see supporting information for details). As a sensitivity analysis, we assessed whether there was a delayed relationship between the virus proxy and outcome variables, including a potential sequential pattern of infection across age groups,^23^, ^38^ by specifying lags of up to 3 weeks for the proxy variables.

Analysis was performed in SAS Enterprise Guide Version 7.15 with base SAS Version 9.4.^39^ The SAS GENMOD procedure^40^ and %BOOT and %BOOTCI macros^41^ were used for regression modelling and bootstrapping.

### 2.5 Ethical approval

Ethical approval was acquired from the University of New South Wales HREAP Executive (HC200168).

## 3 RESULTS

### 3.1 Summary characteristics of the data

A total of 2.89 million principal respiratory hospital admissions were recorded during the study period from July 2008 through June 2018. Mean weekly hospitalisation counts were 5549 (range 3112–10,308) respiratory, 3709 (range 1931–8044) ARI, 1525 (range 83–4425) and 344 (range 66–760) bronchiolitis (in <5‐year‐olds only) (Table S10). During the same period, there were 73,019 (80.7% of the all‐age total) RSV‐coded and 14,245 (15.4% of the all‐age total) influenza‐coded hospital admissions in children aged <5 years, considering both principal and other admission diagnoses. Further detail on age‐specific observed hospitalisations can be found in the supporting information.

**FIGURE 1:**
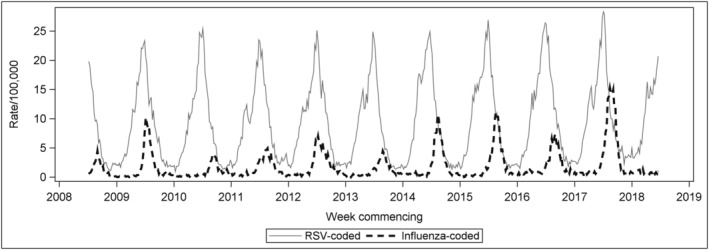
Weekly rate per 100,000 population of RSV‐coded and influenza‐coded hospitalisations (principal or other diagnosis field) for children aged <5 years, Australia, July 2008 to June 2018. RSV, respiratory syncytial virus

Figure 1 shows the weekly rate per 100,000 population of RSV‐ and influenza‐coded hospitalisations for children aged <5 years, which is clearly seasonal. For RSV, the annual pattern was similar in all years, with a slightly increasing trend and peaking mid‐winter (around July). For influenza, the peak occurred from late winter to early spring, and epidemic magnitude varied but also showed an increasing trend. Influenza epidemics also appeared to follow the peak of RSV activity (Figure 1).

### 3.2 Model assessment and sensitivity analysis

Generally, the models fitted the observed data relatively well, with slight overfitting and underfitting in some years of the study period (see supporting information for detail on model assessment). In the sensitivity analysis, lagging of the viral activity proxies did not substantially improve model performance (results not shown); thus, it was not incorporated into the final model.

### 3.3 Estimates of the average annual and annual RSV‐ and seasonal influenza‐attributable hospitalisations for the period considered

The estimated average annual hospitalisation counts and rates per 100,000 population by virus, outcome of interest and age group from 2009 to 2017 (excluding 2009 for seasonal influenza) are shown in Table 1. The all‐age rate of respiratory hospitalisation attributable to RSV was 54.8 (95% CI: 20.1, 88.8) per 100,000 population, which was relatively similar to that of ARI and about fourfold that of the P&I. The all‐age rate of hospitalisation for seasonal influenza was 87.8 (95% CI: 74.5, 97.7) per 100,000 population, around 62% higher than that of the RSV. Seasonal influenza‐attributable hospitalisation rates (for all ages combined) were higher than that of RSV across all applicable outcomes on average, most notable in the P&I category with a rate almost 3.5 times higher than that of RSV. Overall, RSV‐attributable respiratory hospitalisations were estimated to be approximately eight times that estimate for seasonal influenza in <5‐year‐olds but around one third times that estimate for seasonal influenza in ≥5‐year‐olds.

**TABLE 1:** Estimated average annual RSV‐ and seasonal^a^ influenza‐attributable hospitalisation rates per 100,000 population and counts (95% confidence intervals), by principal diagnosis and age group, from 2009 to 2017 for RSV and 2010 to 2017 for seasonal influenza, Australia

Principal diagnosis		Age group (in years)						
		0–4	5–14	15–44	45–64	65–74	≥75	All ages^b^
		**Rate**						
Bronchiolitis	RSV	466.9 (418.2, 525.4)	n/a	n/a	n/a	n/a	n/a	n/a
	Seasonal influenza	−17.5 (−33.6, −4.7)	n/a	n/a	n/a	n/a	n/a	n/a
P&I	RSV	87.2 (45.5, 127.1)	4.3 (−12.7, 21.6)	3.0 (−6.8, 12.6)	−6.8 (−19.6, 5.8)	−4.9 (−44.9, 32.3)	128.0 (−26.8, 291.9)	13.6 (−2.2, 29.3)
	Seasonal influenza	89.4 (76.2, 100.3)	29.5 (24.5, 34.8)	25.9 (22.6, 28.8)	45.8 (41.5, 49.2)	88.9 (74.5, 100.2)	326.2 (270.1, 375.0)	60.0 (53.7, 64.4)
ARI	RSV	589.8 (466.8, 719.9)	−3.6 (−30.2, 24.0)	3.8 (−10.3, 17.5)	3.5 (−17.7, 23.9)	33.5 (−31.9, 103.8)	256.4 (5.3, 487.4)	59.7 (31.7, 86.2)
	Seasonal influenza	95.1 (57.2, 129.1)	41.3 (32.9, 50.0)	31.8 (27.1, 36.0)	68.9 (61.5, 75.0)	150.3 (125.0, 171.4)	483.3 (397.5, 550.4)	85.2 (147.4, 179.8)
Respiratory	RSV	464.2 (285.9, 641.2)	−72.3 (−128.0, −19.1)	7.2 (−13.8, 28.3)	8.8 (−21.9, 38.5)	64.4 (−16.7, 153.8)	359.7 (79.0, 627.5)	54.8 (20.1, 88.8)
	Seasonal influenza	57.5 (5.3, 107.7)	26.3 (10.4, 43.9)	33.9 (26.7, 40.5)	78.1 (67.6, 87.2)	165.2 (135.1, 190.5)	521.6 (420.9, 600.0)	87.8 (74.5, 97.7)
		**Count**						
Bronchiolitis	RSV	7017 (6285, 7820)	n/a	n/a	n/a	n/a	n/a	n/a
	Seasonal influenza	−255 (−497, −69)	n/a	n/a	n/a	n/a	n/a	n/a
P&I	RSV	1321 (689, 1907)	125 (−365, 614)	290 (−661, 1208)	−390 (−1122, 331)	−91 (−836, 634)	1903 (−446, 4293)	3159 (−501, 6720)
	Seasonal influenza	1373 (1184, 1540)	862 (724, 1016)	2543 (2248, 2827)	2664 (2441, 2859)	1732 (1467, 1951)	4981 (4174, 5725)	14,155 (12,842, 15,189)
ARI	RSV	8934 (7069, 10,799)	−104 (−868, 682)	373 (−1000, 1677)	199 (−1013, 1357)	625 (−594, 1914)	3810 (79, 7169)	13,837 (7345, 19,747)
	Seasonal influenza	1461 (889, 1982)	1206 (974, 1461)	3124 (2699, 3535)	4004 (3617, 4363)	2927 (2462, 3338)	7380 (6143, 8404)	20 102 (17,909, 21,849)
Respiratory	RSV	7031 (4331, 9618)	−2081 (−3682, −544)	698 (−1338, 2713)	505 (−1253, 2183)	1199 (−312, 2836)	5346 (1175, 9230)	12,698 (4679, 20,334)
	Seasonal influenza	883 (82, 1654)	768 (307, 1283)	3330 (2656, 3983)	4540 (3975, 5072)	3217 (2662, 3710)	7965 (6505, 9161)	20,702 (17,851, 23,025)

Abbreviations: ARI, acute respiratory infection; n/a, not available; P&I, pneumonia and influenza; RSV, respiratory syncytial virus.

^a^ Calculation for the annual average seasonal influenza‐attributable hospitalisation excluded the pandemic year 2009.

^b^ The rate refers to the crude rate. Calculation for the all‐ages estimates included both significant and non‐significant values.

The RSV‐attributable hospitalisation burden varied by age group with the highest estimated average annual RSV‐attributable respiratory hospitalisation rate of 464.2 per 100,000 (95% CI: 285.9, 641.2) in children <5 years of age. The rate declined as age increases until the older age group of 65 years and above, where RSV‐attributable rates started to rise again. A similar pattern was seen with the outcome of ARI hospitalisations, with the highest rate of 589.8 (95% CI: 466.8, 719.9) in children <5 years of age. However, for P&I, the highest rate of RSV‐attributable hospitalisation was seen in ≥75‐year‐olds, with an estimated average annual rate of 128.0 (95% CI: −26.8, 291.9). While a high rate of RSV‐attributable P&I hospitalisation was also observed in <5‐year‐olds, relatively lower rates were found in persons aged between 5 and 74 years. Lastly, the estimated rate of RSV‐attributable bronchiolitis hospitalisation in children aged <5 years was 466.9 (95% CI: 418.2, 525.4), similar to those of the ARI and respiratory diagnostic categories (Table 1).

The highest average annual estimated seasonal influenza‐attributable respiratory hospitalisation rate was 521.6 (95% CI: 420.9, 600.0), which occurred in persons aged ≥75 years old. This rate per 100,000 decreased with age groups from 65–74 to 5–14 years old and slightly increased in children <5 years. Seasonal influenza‐attributable ARI and P&I hospitalisation rates were also highest in persons of age ≥ 75 years. Consistent with the respiratory outcome, relatively lower but statistically significant rates were observed in the other age groups. For the <5‐year‐olds, high estimates of seasonal influenza‐attributable rates of ARI and P&I hospitalisations were found. In children aged <5 years, the seasonal influenza‐attributable bronchiolitis hospitalisation rate estimate was negative (Table 1).

Estimates of seasonal influenza‐attributable hospitalisations varied sustainably from 2010 through 2017, with less yearly variation in the estimated annual RSV‐attributable respiratory hospitalisations. In 2017, the seasonal influenza‐attributable hospitalisations were approximately double the annual average in all age groups. Additional results on the annual RSV‐ and influenza‐attributable hospitalisations are discussed in the supporting information and summarised in Tables S1–S8.

### 3.4 Underestimation of RSV‐ and seasonal influenza‐specific hospitalisations based on hospital diagnosis codes

**FIGURE 2:**
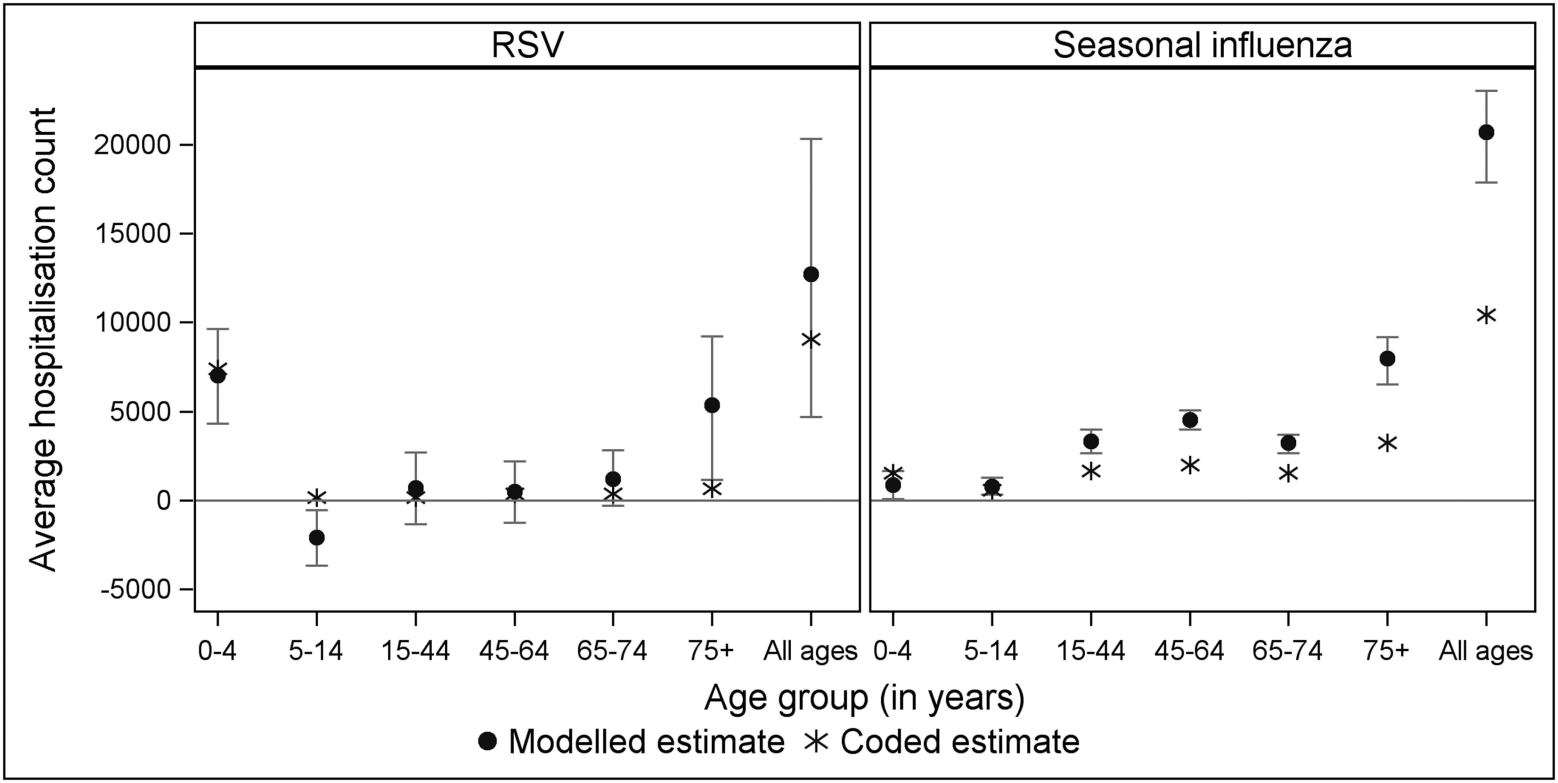
Comparison of the average annual estimate of modelled (attributable) and coded hospitalisations (any diagnosis field) from 2009 to 2017 for RSV and 2010 to 2017 for seasonal influenza, by age group, Australia. RSV, respiratory syncytial virus

For RSV, underestimation of hospitalisation burden when using hospital diagnosis codes alone was most evident in ≥75‐year‐olds, where the modelled estimate was around eightfold higher than the coded estimate (Figure 2; Table S11). In younger age groups, modelled and coded estimates of RSV hospitalisations were less distinct, with very similar estimates in those <5 years of age and relatively low estimates for both modelled and coded in younger adults. For seasonal influenza, coded hospitalisations were significantly lower than model estimated hospitalisations in all age groups ≥15 years (Figure 2).

## 4 DISCUSSION

The estimated average, all‐age RSV‐ and seasonal influenza‐attributable respiratory hospitalisation rates were 54.8 and 87.8 per 100,000 population each year in Australia, representing approximately 12,700 and 20,700 average annual hospitalisations, respectively. We found substantial RSV‐attributable respiratory hospitalisations in those aged ≥75 years, which was estimated to be far higher than RSV estimates based solely on hospital diagnosis codes (approximately eight times higher). We also found evidence of significant underestimation of seasonal influenza hospitalisation burden using diagnostic codes across all adult age groupings, which again was highest for those ≥75 years (approximately double the coded estimate). For young children <5 years of age, the rate of respiratory hospitalisation attributable to RSV was higher than the estimate attributable to seasonal influenza. However, for all other age groups, the average annual all‐age attributable rates of hospitalisation were higher for seasonal influenza than for RSV.

Our all‐age average annual estimate of RSV‐attributable rate of respiratory hospitalisation was about twice the recently estimated rate for Australia from 2006 to 2015 (29 per 100,000 population), which was based on diagnostic codes only rather than modelling.^35^ As RSV hospitalisations may be potentially assigned non‐RSV‐specific codes,^42^, ^43^ estimates based on diagnostic codes alone may underestimate the true burden, particularly in older adults. The modelling approach we applied may help overcome the underestimation of RSV disease burden. Our estimate of the average annual rate of all‐age seasonal influenza‐attributable hospitalisation was slightly higher but comparable with the recent estimates in Australia from 2001 to 2013 (77 per 100,000 population)^10^ and from 2007 to 2015 (57 per 100,000 population).^44^ These estimates were obtained using similar modelling approaches to our study but different proxies of influenza activity.

In children aged <5 years, our average annual RSV‐attributable respiratory hospitalisation rate (464 per 100,000) was similar to the reported rates estimated from diagnostic codes alone for 2006–2015 (418 per 100,000 population)^35^ and those estimated in a New South Wales (NSW) cohort study from 2001 to 2010 based on linked data (490 per 100,000 population).^45^ This, alongside our finding of the similarity between coded hospitalisations and modelled estimates in young children, suggests limited hospital underdiagnosis in this age group.

Especially in those aged ≥75 years, our results suggest that counting only hospitalisations that have specific infection codes will substantially underestimate RSV and influenza burden (Figure 2; Table S11). For RSV, modelled estimates were about eight times higher in those aged over 75 years than those based on coded hospitalisation. This exceeded the underestimation for seasonal influenza in this age group, where the modelled estimate was two times higher than coded hospitalisations. Our modelled results for RSV in older adults contrast with previous Australian estimates based on coded RSV hospitalisations alone (6 per 100,000 population in those ≥65 years).^35^ However, although there were differences, overall, our RSV estimates were broadly compatible with estimates found in the United States^18^ and the United Kingdom^23^, ^24^ (Table S12).

Previous Australia studies have reported that the average annual rates of seasonal influenza‐attributable hospitalisation in older adults were 237 per 100,000 population (in ≥85‐year‐olds for 2001–2013)^10^ and 303 per 100,000 population (in ≥75‐year‐olds for 2007–2015).^44^ The relatively higher estimate that we found in those aged over 75 years (522 per 100,000 population) in part reflects the increase in the estimated rate of seasonal influenza‐attributable hospitalisations since 2014, with 2017 being the most severe influenza year for respiratory hospitalisations across all age groups over the study period.

Influenza showed negative attributions for bronchiolitis hospitalisations. This may be due to the absence of influenza as a cause of bronchiolitis^46^ and lack of precision in the modelling. Alternately, because influenza epidemics followed the peak in RSV epidemics, this might represent some unmodelled interference between the two viruses in the population.^47^, ^48^ Our models estimated negative RSV‐attributable respiratory hospitalisations in children aged 5–14 years, a result that has also been observed in other modelling studies.^18^, ^24^ This might be related to the very small attributable RSV hospitalisation burden for the age group,^17^, ^49^ statistical artefact or the non‐specific outcome considered.^18^

One limitation of our study was the use of pathogen‐specific ICD coded hospitalisations from admission records of <5‐year‐olds as proxies for RSV and influenza relative activity, which may not completely represent virus activity in other age groups. However, it was evident that older age groups were affected by markedly increased testing over time.^36^ Whereas diagnostic testing for this younger age group was relatively stable over the study period and as a result may more reasonably reflect the relative activity of RSV and influenza infections in the population over the study period. However, the proxy of the influenza activity might not have fully reflected the relative infection risk in older persons in some years, which may have resulted in slight overestimation of the total outcomes for the ≥75‐year‐olds models in 2009 and underestimation in 2012, 2016 and 2017. Another caveat is that the RSV proxy variable also showed a relatively high correlation with the ‘cosine’ variable of the trigonometric terms, which may indicate a potential reduction to the portion of outcomes attributable to RSV but also reduces the possibility of overestimating the burden. Finally, we could not account for testing and coding practices that may have changed over time or varied across states and territories in Australia.

## 5 CONCLUSIONS

Our study provides important new estimates of the hospitalisation burden for RSV across all age groups and updated estimates of influenza‐attributable hospitalisation in Australia. RSV was found to be a major cause of hospitalisation not only in young children but also among older adults, where substantial underestimation was found. Influenza‐attributable hospitalisations were highest in 2017 and were substantial, particularly in older adults.

## FUNDING INFORMATION

No funding was received to support this specific study.

## CONFLICT OF INTEREST

Hannah Moore is a lead investigator on a Merck Sharp and Dohme Investigator Initiated study and has previously received consultancy payment for participation in expert input forums. These activities are not related to her involvement in this current manuscript. David Muscatello is an unpaid member of the Immunisation Coalition, a not‐for‐profit organisation that delivers science‐based advocacy for immunisation against infectious disease in Australia. The Coalition's revenue sources include healthcare and pharmaceutical companies. All other authors reported no known potential conflicts of interest.

## AUTHOR CONTRIBUTIONS

**Allen Nazareno**: Conceptualisation; formal analysis; methodology; validation; software; writing—original draft preparation; writing—review and editing. **David Muscatello**: Conceptualisation; project administration; methodology; supervision; writing—review and editing. **Robin Turner**: Methodology; supervision; writing—review and editing. **James Wood**: Conceptualisation; methodology; supervision; writing—review and editing. **Hannah Moore**: Methodology; supervision; writing—review and editing. **Anthony Newall**: Conceptualisation; project administration; methodology; supervision; writing—review and editing.

## Supporting information


**Figure S1.** Flowchart of the bootstrapping process in calculating the confidence intervals (methods adopted from the work of Goldstein et al^2^)
**Figure S2.** Autocorrelation and partial autocorrelation plots of the residuals of the pneumonia and influenza, age ≥75 years model.
**Figure S3.** Autocorrelation and partial autocorrelation plots of the residuals of the bronchiolitis, age <5 years model.
**Figure S4.** Observed, baseline, and estimated RSV‐attributable and influenza‐attributable respiratory hospitalisation rates per 100 000 population by age group, Australia, 2009–2017.
**Figure S5.** Observed, baseline, and estimated RSV‐attributable and influenza‐attributable acute respiratory infection (ARI) hospitalisation rates per 100 000 population by age group, Australia, 2009–2017.
**Figure S6.** Observed, baseline, and estimated RSV‐attributable and influenza‐attributable pneumonia and influenza (P&I) hospitalisation rates per 100 000 population by age group, Australia, 2009–2017.
**Figure S7.** Observed, baseline, and estimated RSV‐attributable and influenza‐attributable bronchiolitis hospitalisation rates per 100 000 population, children aged <5 years, Australia, 2009–2017.
**Table S1.** Estimated annual and average rate (95% confidence interval) per 100 000 population of respiratory hospitalisations attributable to RSV and influenza, Australia, 2009–2017
**Table S2.** Estimated annual and average rate (95% confidence interval) per 100 000 population of acute respiratory infection (ARI) hospitalisations attributable to RSV and influenza, Australia, 2009–2017
**Table S3.** Estimated annual and average rate (95% confidence interval) per 100 000 population of pneumonia and influenza (P&I) hospitalisations attributable to RSV and influenza, Australia, 2009–2017
**Table S4.** Estimated annual and average rate (95% confidence interval) per 100 000 population of bronchiolitis hospitalisations attributable to RSV and influenza, Australia, 2009–2017
**Table S5.** Estimated annual and average (95% confidence interval) number of respiratory hospitalisations attributable to RSV and influenza, Australia, 2009–2017
**Table S6.** Estimated annual and average (95% confidence interval) number of acute respiratory infection (ARI) hospitalisations attributable to RSV and influenza, Australia, 2009–2017
**Table S7.** Estimated annual and average (95% confidence interval) number of pneumonia and influenza (P&I) hospitalisations attributable to RSV and influenza, Australia, 2009–2017
**Table S8.** Estimated annual and average (95% confidence interval) number of bronchiolitis hospitalisations attributable to RSV and influenza, Australia, 2009–2017
**Table S9.** Estimated age‐standardised all‐age rates of hospitalisation (95% confidence interval) attributable to RSV and influenza, by diagnostic category and year, Australia, 2009–2017
**Table S10.** Summary statistics for the weekly number of hospitalisations (principal diagnosis) for each diagnostic category, Australia, 1 July 2008–30 June 2018^†^
**Table S11.** RSV and influenza‐coded respiratory hospitalisations (any diagnosis field), Australia, 2009–2017^†^
**Table S12.** Comparison of selected studies of RSV‐ and influenza‐attributable respiratory hospitalisations (rate per 100 000 population) from the United Kingdom (UK) and United States (US)

## Data Availability

The hospitalisation data used in the study were provided by the AIHW and cannot be made available by the authors upon request.
