# Supplementary material for: Modelled estimates of hospitalisations attributable to respiratory syncytial virus and influenza in Australia, 2009–2017
Source: Influenza Other Respir Viruses. 2022 Jun 30;16(6):1082–90. doi: 10.1111/irv.13003 (PMC9530581; doi:10.1111/irv.13003)
Supplement: Supplementary file 1 — Figure S1. Flowchart of the bootstrapping process in calculating the confidence intervals (methods adopted from the work of Goldstein et al2) Figure S2. Autocorrelation and partial autocorrelation plots of the residuals of the pneumonia and influenza, age ≥75 years model. Figure S3. Autocorrelation and partial autocorrelation plots of the residuals of the bronchiolitis, age <5 years model. Figure S4. Observed, baseline, and estimated RSV‐attributable and influenza‐attributable respiratory hospitalisation rates per 100 000 population by age group, Australia, 2009–2017. Figure S5. Observed, baseline, and estimated RSV‐attributable and influenza‐attributable acute respiratory infection (ARI) hospitalisation rates per 100 000 population by age group, Australia, 2009–2017. Figure S6. Observed, baseline, and estimated RSV‐attributable and influenza‐attributable pneumonia and influenza (P&I) hospitalisation rates per 100 000 population by age group, Australia, 2009–2017. Figure S7. Observed, baseline, and estimated RSV‐attributable and influenza‐attributable bronchiolitis hospitalisation rates per 100 000 population, children aged <5 years, Australia, 2009–2017. Table S1. Estimated annual and average rate (95% confidence interval) per 100 000 population of respiratory hospitalisations attributable to RSV and influenza, Australia, 2009–2017 Table S2. Estimated annual and average rate (95% confidence interval) per 100 000 population of acute respiratory infection (ARI) hospitalisations attributable to RSV and influenza, Australia, 2009–2017 Table S3. Estimated annual and average rate (95% confidence interval) per 100 000 population of pneumonia and influenza (P&I) hospitalisations attributable to RSV and influenza, Australia, 2009–2017 Table S4. Estimated annual and average rate (95% confidence interval) per 100 000 population of bronchiolitis hospitalisations attributable to RSV and influenza, Australia, 2009–2017 Table S5. Estimated annual and average (95% confidence inter [file IRV-16-1082-s001.docx]

**Modelled estimates of hospitalisations attributable to respiratory syncytial virus and influenza in Australia, 2009-2017**

**Supplementary information**

**Supplementary information related to the Methods**

**Statistical modelling**

*Holiday variables*

Holiday variables incorporated into the model, as considered the work of Moa et al.^1^ are *week before Christmas*, *Christmas week*, *New year week*, *one week after New year*, *two weeks after New year*, *three weeks after New year*, *Australia day week*, *one week before Easter*, *Easter week*, and *ANZAC day week*, indexed from 6 to 15 (referring to the model formulation), respectively. Each holiday variable was formed by setting the value for a given holiday week to 1 and 0 otherwise each year.

*Calculation of crude and age-standardised hospitalisation rate*

The crude weekly all-age RSV- and influenza-attributable rates of hospitalisation were calculated as follows:

$$Crude rate_{all-age}^{j}=\frac{\sum_{i=1}^{n} Weekly count_{i}^{j}}{Total population}$$

where $Weekly count_{i}^{j}$ is the weekly number of hospitalisations attributable to a virus $j$ for age group $i$, and $Total population$ is the weekly total population estimate.

Apart from the crude rate, age-standardised weekly virus-attributable all-age hospitalisation rates were computed by getting the weighted average of hospitalisation rates, expressed mathematically as:

$$Age-standardised rate_{all-age}^{j}=\sum_{i=1}^{n} \left( \hat{Pop}_{i}\times Weekly rate_{i}^{j} \right)$$

where $\hat{Pop}_{i}$ is the proportion of people in age group $i$ in a standard population (2018 mid-year population estimate), $Weekly rate_{i}$ is the weekly hospitalisation rate attributable to the virus $j$ for each age group $i$.

**Model assessment and sensitivity analysis**

Autocorrelation in the residuals has been identified as a problem in previous analyses,^2,3^ leading to potentially incorrect confidence bounds for the model parameter estimates. To address the autocorrelation problem, the 95% confidence intervals (CIs) of the estimated annual and average annual hospitalisations attributable to RSV and influenza were obtained using a similar bootstrapping approach applied by Goldstein et al.^2^ (Supplementary Figure 1).

**Supplementary Figure 1. Flowchart of the bootstrapping process in calculating the confidence intervals (methods adopted from the work of Goldstein et al.^2^**)


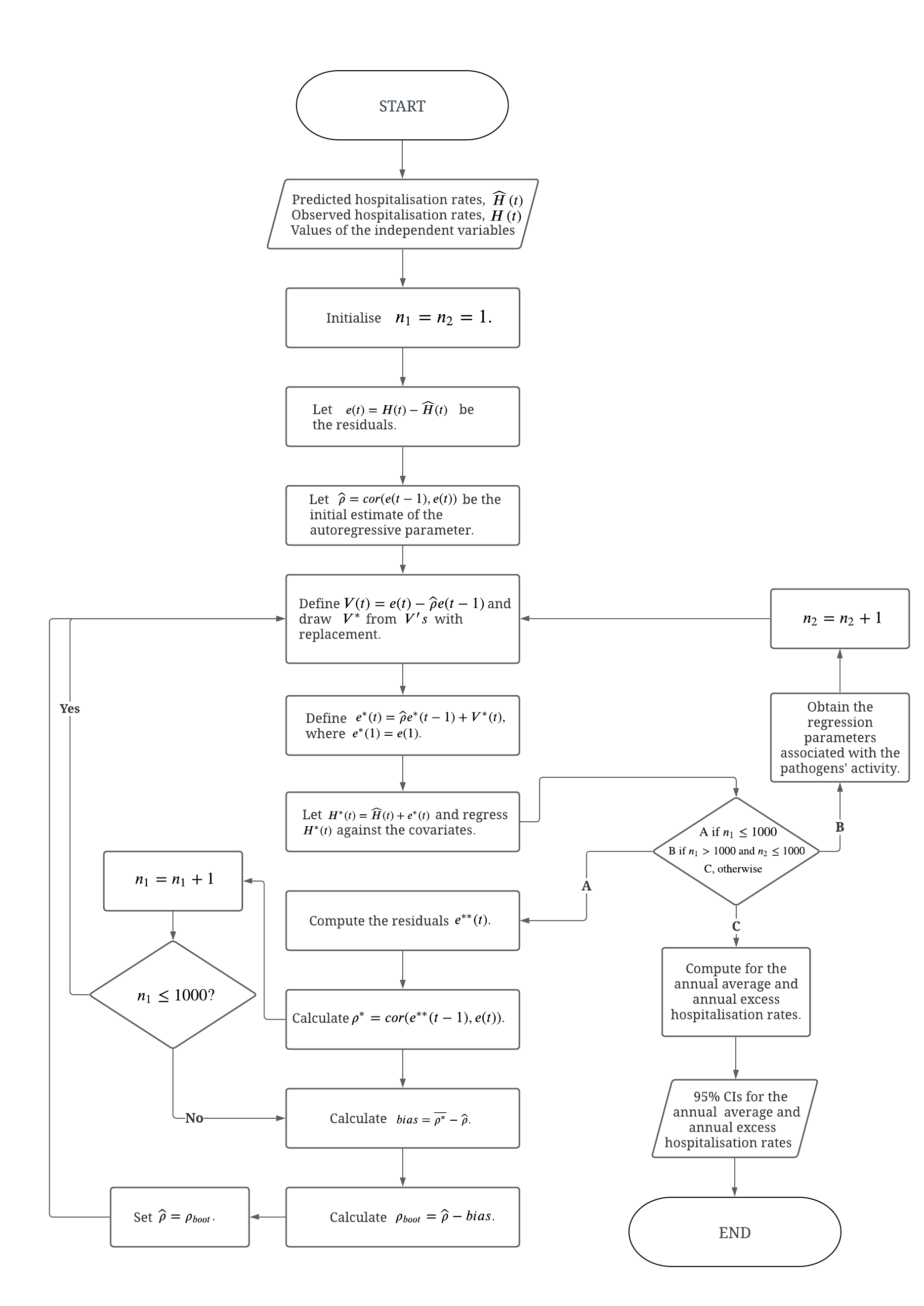


The different shapes in the flowchart above are described as follows: 1) Ovals called as terminators represent the beginning or end of the procedure; 2) rectangles indicate the different processes; 3) parallelograms are the inputs or outputs; 4) the diamonds symbolise the various decisions; and 5) arrows show the direction or flow of the procedure.

The bootstrapping process involves two main steps: 1) obtaining a bootstrap estimate of the autoregressive parameter and 2) obtaining a bootstrap estimate of annual and average annual excess hospitalisation rate. For the first step, the residuals, $e$, are modelled as an autoregressive (AR) model of order 1 (provided adequate) using the initial estimate of the AR parameter $\hat{\rho}$. The parameter $\hat{\rho}$ is obtained by taking the autocorrelation between $e\left( t \right)$ and $e(t-1)$. As this estimate tends to be underestimated, the bias is calculated using the bootstrap method. The bootstrap procedure starts with resampling (with replacement) the AR residuals, $V$ to form $V^{*}$. The residuals (of the original model) are recreated using the formula, $e^{*}\left( t \right)=\hat{\rho}e^{*}\left( t-1 \right)+V^{*}(t)$, where $e^{*}\left( 1 \right)=e\left( 1 \right).$ These residuals are added back to the predicted values of $H$ and then regressed against the covariates. The autocorrelation between the residuals of the regression model is obtained as an estimate of the AR parameter, denoted by $\rho^{*}$. The entire process is repeated for 1000 times, creating a sampling distribution of the AR parameter. The initial estimate of the AR parameter is subtracted from mean of $\rho^{*}$, represented as $\bar{\rho^{*}}$, to estimate the bias. The bias is then subtracted from $\hat{\rho}$ and the difference gives the bootstrapped estimate of AR parameter called $\rho_{boot}$.

The second step involves the same procedure as the first but using $\rho_{boot}$ as the estimate for the AR parameter. Moreover, instead of obtaining an AR parameter for each model that would be established, a sample of the regression parameter estimates associated with the RSV and flu activity are obtained. The process is replicated for 1000 times. The parameters are then used to determine the contribution of RSV and flu to hospital admission, which creates a sampling distribution for the excess RSV and influenza-attributed hospitalisation rates used to obtain the 95% confidence bounds on them. Additional details of the approach can be found in the study of Goldstein et al..^2^

**Supplementary information related to the Results**

**Summary characteristics of the data**

For outcomes considered in all age groups, the highest weekly mean hospitalisation rate was observed in persons aged 75 years and above, with a rate of 38.5, 73.7, and 104.2 per 100,000 population in P&I, ARI, and respiratory categories, respectively. Outside of those aged 75 years and above, observed weekly hospitalisation rates in persons aged 65-74 years and 0-4 years were relatively higher than that of the other age groups across the three outcomes. The highest weekly mean hospitalisation rate in <5 year-olds bronchiolitis category was 22.6 per 100,000 population.

**Model assessment and sensitivity analysis**

Autocorrelation plots indicated autocorrelation in the model residuals (see Supplementary Figures 2 and 3 for illustrative examples). Further, the results of the correlation analysis between RSV and influenza proxy variables showed that RSV and influenza were weakly correlated, with a correlation coefficient of 0.23. Also, the RSV proxy variable had a weak negative correlation (Pearson correlation coefficient: -0.26) with the sine term but a strong linear relation with the cosine term (Pearson correlation coefficient: 0.89).

**Supplementary Figure 2. Autocorrelation and partial autocorrelation plots of the residuals of the pneumonia and influenza, age ≥75 years model.**


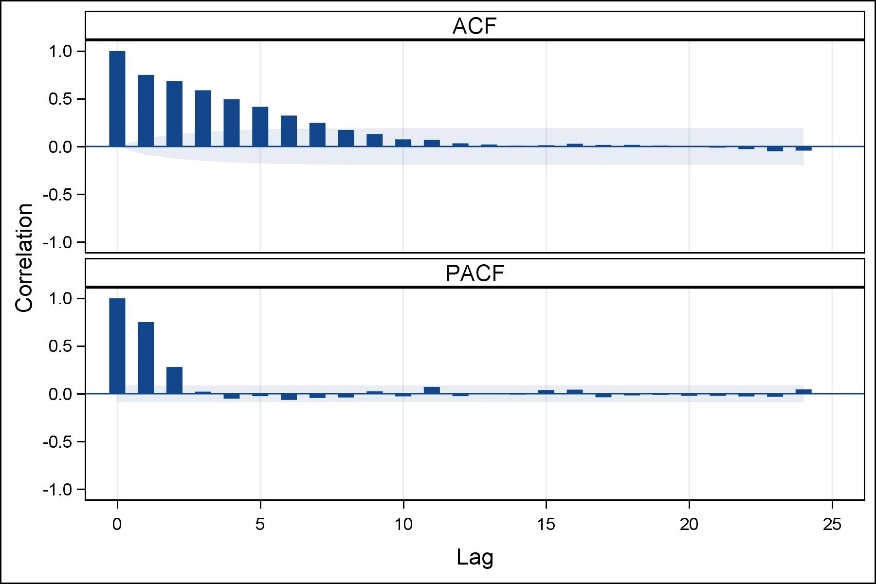


**Supplementary Figure 3. Autocorrelation and partial autocorrelation plots of the residuals of the bronchiolitis, age <5 years model.**


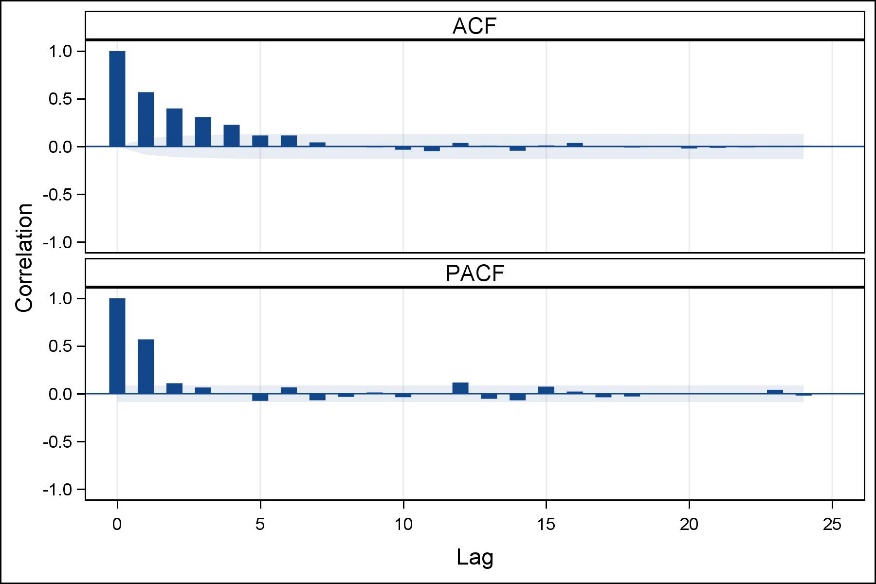


**Estimates of the annual RSV- and influenza-attributable hospitalisations for the different years considered**

The time series of the estimated age-specific RSV- and influenza-attributable hospitalisation rates per 100,00 population over our estimated baseline level are presented in Supplementary Figures 4-7. Supplementary Tables 1-8 provide annual estimates of RSV and influenza-attributable hospitalisations. Overall, the rates of RSV-attributable respiratory hospitalisations for all ages combined were relatively consistent over the years, with the highest rate of 62.6 (95% CI: 22.9, 100.7) in 2016. The estimated all-age RSV-attributable hospitalisation rates for ARI and P&I showed similar patterns in terms of consistency of estimates between years.

Unlike RSV, the pattern for seasonal influenza-attributable hospitalisations was not consistent between years for all outcomes and age groups. The highest all-age rate of seasonal influenza-attributable hospitalisation was recorded in 2017, about twice the average rate in the previous 5 years. The all-age rate of influenza-attributable respiratory hospitalisation in 2017 was 170.6 (95% CI: 147.2, 189.7) per 100,000 population. Influenza-attributable hospitalisation rates were also higher in 2014 through 2016, above the estimate for the pandemic year (2009). The between year trends of the influenza-attributable hospitalisations for both ARI and P&I were the same as that of the respiratory, with the highest estimated annual rates recorded in 2017.

For all the outcomes, RSV was consistently associated with hospitalisations in children <5 years of age (as indicated by the red shade) from 2009-2017. For bronchiolitis, hospitalisations in <5 year-olds were predominantly attributed to RSV. Excluding the bronchiolitis outcome, a substantial rate of RSV-attributable hospitalisations was seen among the ≥75 year-olds. In influenza, the attributed hospitalisation rate was substantial but varying over the years for all the age groups (as indicated by the blue shade), with a more pronounced magnitude in persons aged 65-74 and ≥75 years.

**Supplementary Figure 4. Observed, baseline, and estimated RSV-attributable and influenza-attributable respiratory hospitalisation rates per 100,000 population by age group, Australia, 2009-2017.**


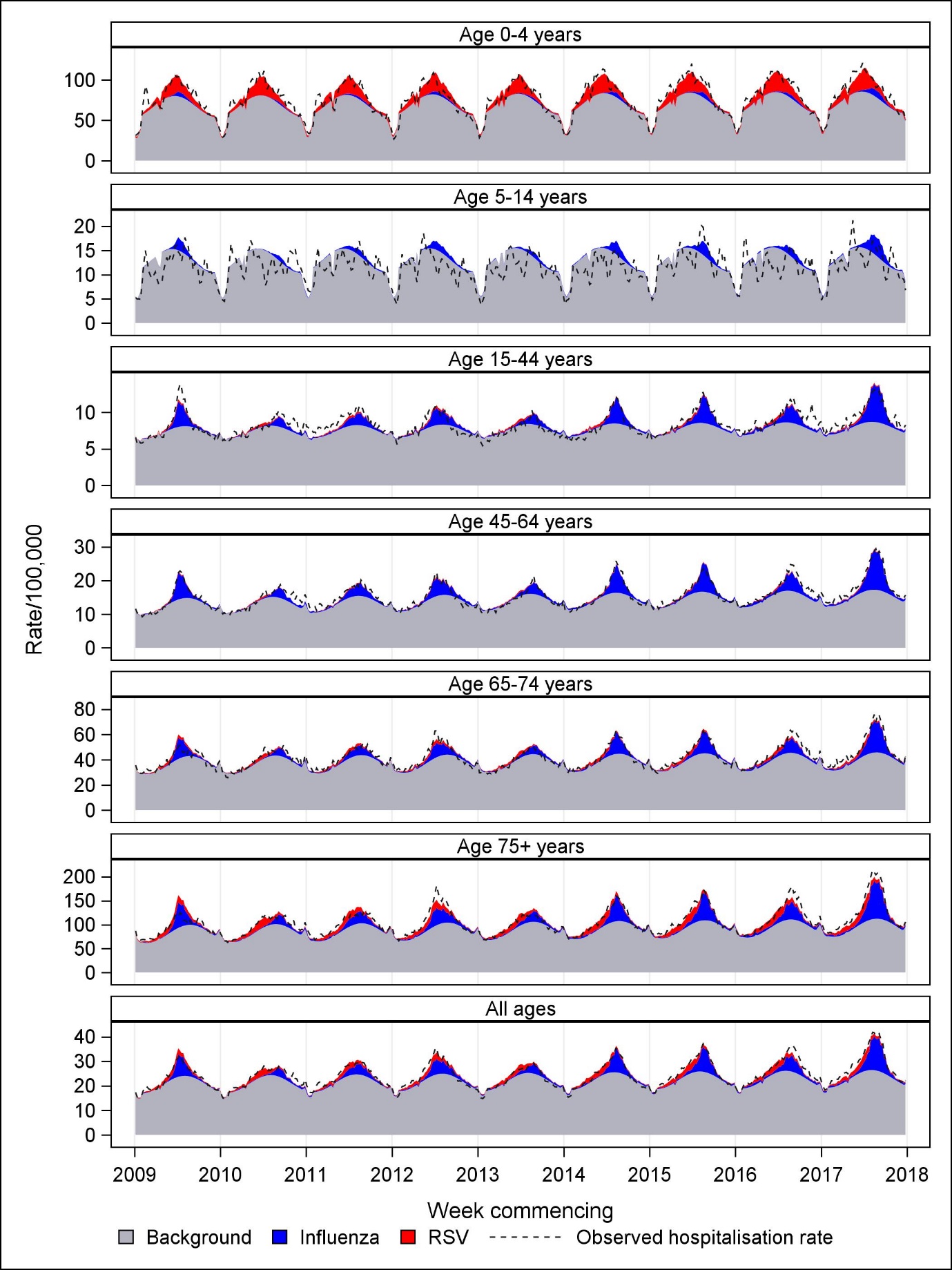


**Supplementary Figure 5. Observed, baseline, and estimated RSV-attributable and influenza-attributable acute respiratory infection (ARI) hospitalisation rates per 100,000 population by age group, Australia, 2009-2017.**


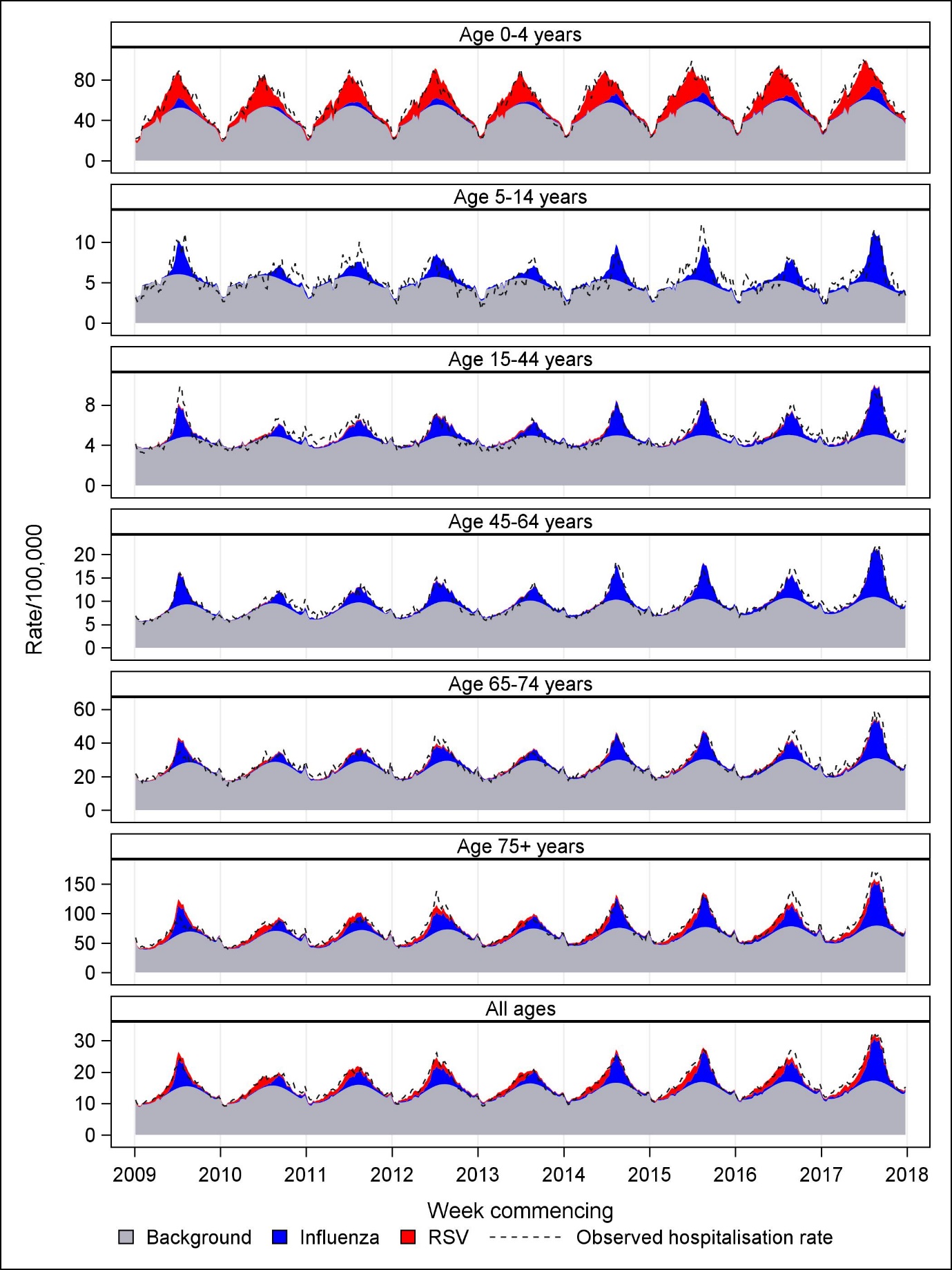


**Supplementary Figure 6. Observed, baseline, and estimated RSV-attributable and influenza-attributable pneumonia and influenza (P&I) hospitalisation rates per 100,000 population by age group, Australia, 2009-2017.**


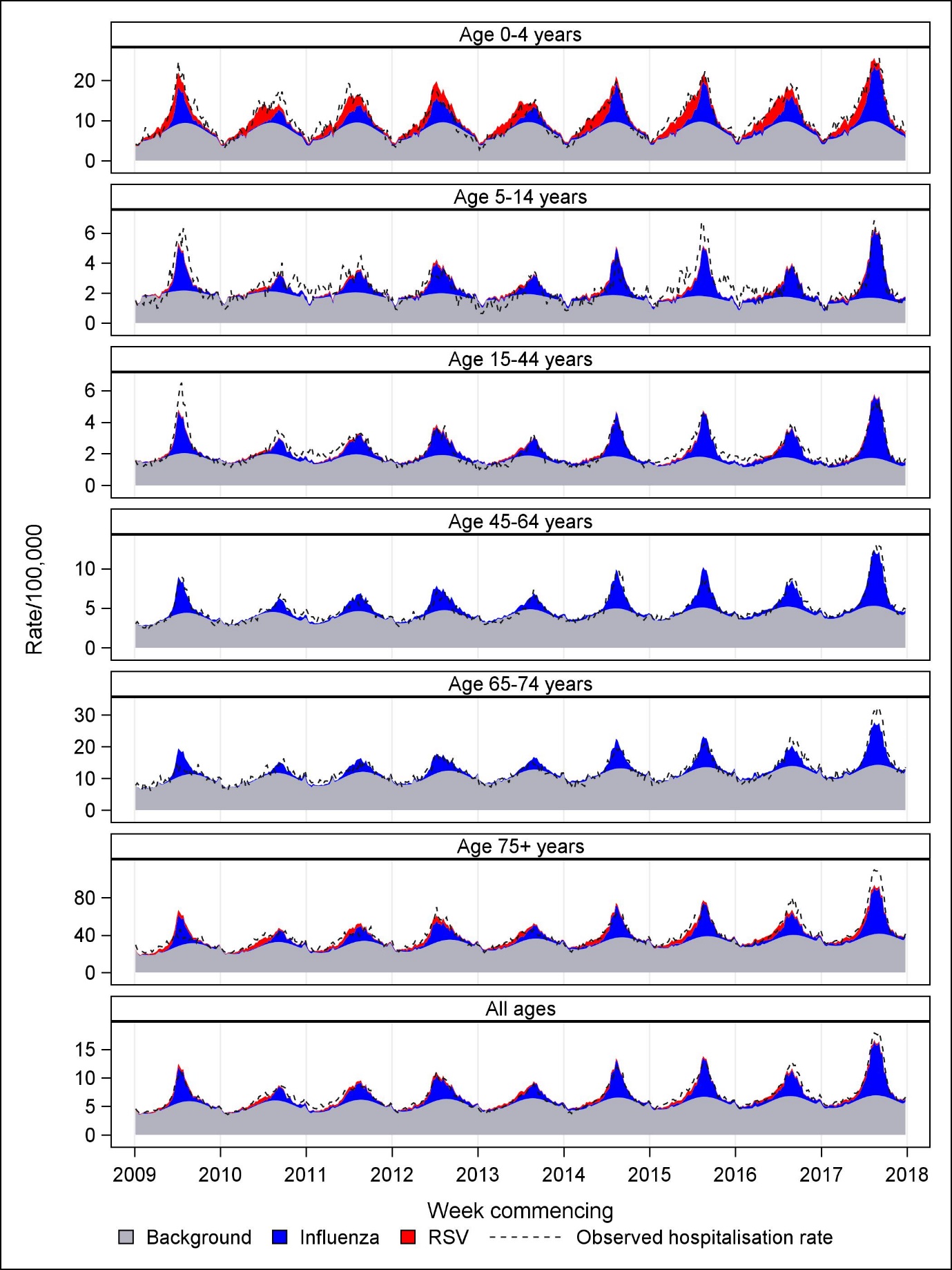


**Supplementary Figure 7. Observed, baseline, and estimated RSV-attributable and influenza-attributable bronchiolitis hospitalisation rates per 100,000 population, children aged <5 years, Australia, 2009-2017.**


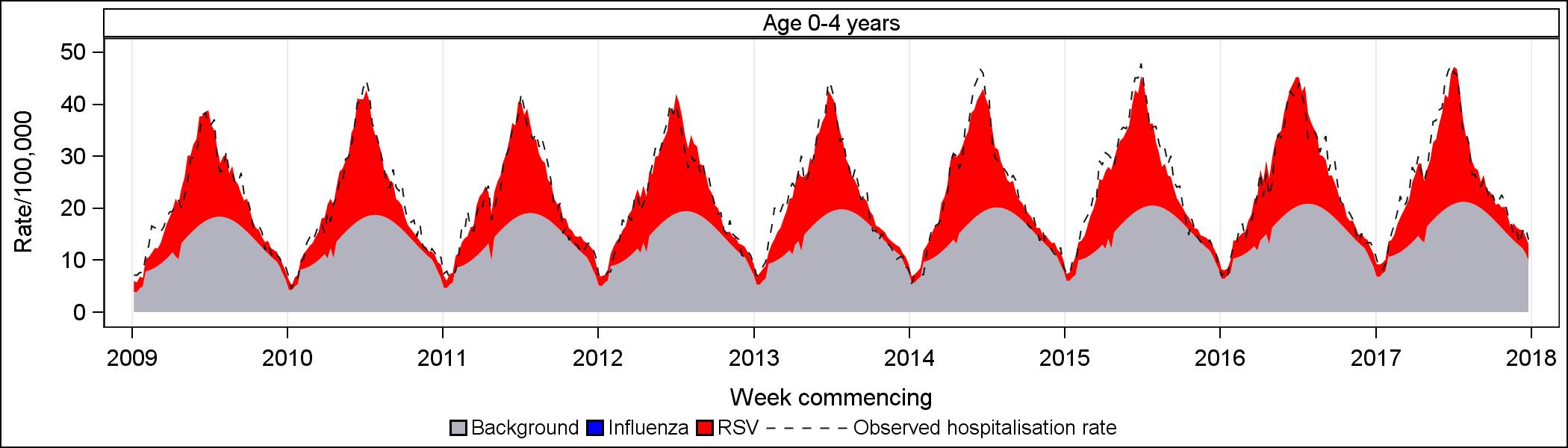


**Supplementary Table 1. Estimated annual and average rate (95% confidence interval) per 100,000 population of respiratory hospitalisations attributable to RSV and influenza, Australia, 2009-2017**

|  | **Age 0 to 4 years** | | **Age 5 to 14 years** | | **Age 15 to 44 years** | | **Age 45 to 64 years** | | **Age 65 to 74 years** | | **Age ≥75 years** | | **All ages**^‡^ | |
| --- | --- | --- | --- | --- | --- | --- | --- | --- | --- | --- | --- | --- | --- | --- |
| **Year** | **RSV** | **Influenza** | **RSV** | **Influenza** | **RSV** | **Influenza** | **RSV** | **Influenza** | **RSV** | **Influenza** | **RSV** | **Influenza** | **RSV** | **Influenza** |
| 2009 | 428.1  (263.7, 585.6) | 51.9  (4.8, 97.1) | -66.1  (-116.9, -17.3) | 23.4  (9.3, 39.1) | 6.6  (-12.7, 25.7) | 30.4  (24.2, 36.3) | 8.1  (-20.1, 35.0) | 70.1  (61.4, 78.4) | 59.2  (-15.4, 140.0) | 148.2  (122.6, 170.9) | 329.6  (72.4, 569.1) | 465.7  (380.4, 535.7) | 49.3  (18.0, 78.7) | 76.2  (65.6, 85.1) |
| 2010 | 428.2  (263.8, 585.9) | 26.1  (2.4, 48.9) | -66.9  (-118.3, -17.5) | 11.9  (4.8, 19.9) | 6.7  (-12.8, 25.9) | 15.4  (12.3, 18.4) | 8.2  (-20.3, 35.3) | 35.6  (31.2, 39.8) | 59.7  (-15.5, 141.1) | 75.5  (62.5, 87.1) | 332.7  (73.1, 574.5) | 237.3  (193.8, 273.0) | 49.9  (18.3, 79.7) | 39.0  (33.6, 43.5) |
| 2011 | 450.3  (277.4, 616.0) | 43.4  (4.0, 81.3) | -69.6  (-123.1, -18.2) | 19.6  (7.8, 32.7) | 6.9  (-13.3, 27.0) | 25.4  (20.2, 30.4) | 8.5  (-21.0, 36.6) | 58.3  (51.0, 65.1) | 62.5  (-16.2, 147.8) | 124.6  (103.1, 143.7) | 346.0  (76.0, 597.4) | 388.9  (317.6, 447.4) | 52.3  (19.1, 83.5) | 64.4  (55.5, 71.8) |
| 2012 | 468.0  (288.3, 640.2) | 59.0  (5.4, 110.5) | -72.7  (-128.5, -19.0) | 26.8  (10.7, 44.7) | 7.2  (-13.9, 28.1) | 34.6  (27.6, 41.3) | 8.8  (-21.9, 38.2) | 79.5  (69.7, 88.9) | 64.8  (-16.8, 153.2) | 168.8  (139.7, 194.7) | 360.7  (79.3, 622.8) | 530.4  (433.1, 610.0) | 54.9  (20.2, 87.6) | 88.2  (76.0, 98.3) |
| 2013 | 418.0  (257.5, 571.9) | 35.0  (3.2, 65.4) | -65.1  (-115.3, -17.0) | 15.9  (6.4, 26.6) | 6.5  (-12.4, 25.2) | 20.5  (16.4, 24.6) | 7.9  (-19.7, 34.3) | 47.4  (41.5, 52.9) | 57.9  (-15.1, 136.9) | 100.3  (83.0, 115.6) | 323.8  (71.2, 559.0) | 316.4  (258.4, 363.9) | 49.4  (18.3, 79.0) | 52.8  (45.5, 58.7) |
| 2014 | 457.8  (282.0, 626.2) | 61.3  (5.7, 114.8) | -71.5  (-126.5, -18.7) | 28.0  (11.2, 46.8) | 7.1  (-13.6, 27.6) | 36.1  (28.8, 43.1) | 8.7  (-21.6, 37.6) | 83.2  (72.9, 93.0) | 63.5  (-16.5, 150.1) | 176.0  (145.6, 203.0) | 355.0  (78.0, 612.9) | 555.5  (453.7, 639.0) | 54.4  (20.1, 87.0) | 93.3  (80.4, 103.8) |
| 2015 | 496.2  (305.7, 678.9) | 67.4  (6.2, 126.2) | -77.3  (-136.8, -20.2) | 30.7  (12.3, 51.3) | 7.7  (-14.7, 29.8) | 39.5  (31.5, 47.3) | 9.4  (-23.4, 40.7) | 91.3  (80.0, 102.0) | 68.6  (-17.8, 162.3) | 192.9  (159.6, 222.5) | 384.1  (84.4, 663.1) | 609.4  (497.7, 701.0) | 59.0  (21.8, 94.6) | 102.9  (88.8, 114.5) |
| 2016 | 522.2  (321.7, 714.4) | 58.8  (5.4, 110.0) | -81.9  (-144.8, -21.4) | 27.0  (10.8, 45.1) | 8.1  (-15.6, 31.6) | 34.7  (27.7, 41.5) | 9.9  (-24.7, 43.0) | 80.1  (70.1, 89.5) | 72.4  (-18.8, 171.3) | 168.5  (139.4, 194.4) | 407.2  (89.5, 703.0) | 535.7  (437.5, 616.2) | 62.6  (22.9, 100.7) | 90.9  (78.4, 101.1) |
| 2017 | 508.9  (313.4, 696.1) | 109.1  (10.1, 204.3) | -80.0  (-141.6, -20.9) | 50.3  (20.1, 84.1) | 8.0  (-15.2, 30.9) | 64.8  (51.7, 77.5) | 9.7  (-24.1, 42.0) | 149.2  (130.6, 166.6) | 70.8  (-18.4, 167.5) | 314.7  (260.4, 363.0) | 397.9  (87.4, 687.0) | 999.2  (816.1, 1149.4) | 61.1  (22.0, 98.5) | 170.6  (147.2, 189.7) |
| **Average**^†^ | **464.2**  **(285.9, 641.2)** | **57.5**  **(5.3, 107.7)** | **-72.3**  **(-128.0, -19.1)** | **26.3**  **(10.4, 43.9)** | **7.2**  **(-13.8, 28.3)** | **33.9**  **(26.7, 40.5)** | **8.8**  **(-21.9, 38.5)** | **78.1**  **(67.6, 87.2)** | **64.4**  **(-16.7, 153.8)** | **165.2**  **(135.1, 190.5)** | **359.7**  **(79.0, 627.5)** | **521.6**  **(420.9, 600.0)** | **54.8**  **(20.1, 88.8)** | **87.8**  **(74.5, 97.7)** |
| Notes:  ^†^Calculation for the annual average seasonal influenza-attributable hospitalisation excluded the pandemic year 2009.  ^‡^Calculation for the all ages estimates included both significant and non-significant values. | | | | | | | | | | | | | | |

**Supplementary Table 2. Estimated annual and average rate (95% confidence interval) per 100,000 population of acute respiratory infection (ARI) hospitalisations attributable to RSV and influenza, Australia, 2009-2017**

|  | **Age 0 to 4 years** | | **Age 5 to 14 years** | | **Age 15 to 44 years** | | **Age 45 to 64 years** | | **Age 65 to 74 years** | | **Age ≥75 years** | | **All ages**^‡^ | |
| --- | --- | --- | --- | --- | --- | --- | --- | --- | --- | --- | --- | --- | --- | --- |
| **Year** | **RSV** | **Influenza** | **RSV** | **Influenza** | **RSV** | **Influenza** | **RSV** | **Influenza** | **RSV** | **Influenza** | **RSV** | **Influenza** | **RSV** | **Influenza** |
| 2009 | 543.9  (430.4, 657.5) | 85.8  (52.2, 116.4) | -3.3  (-27.6, 21.7) | 36.7  (29.7, 44.5) | 3.5  (-9.5, 15.9) | 28.5  (24.6, 32.2) | 3.2  (-16.3, 21.8) | 61.9  (55.9, 67.4) | 30.8  (-29.3, 94.5) | 134.8  (113.4, 153.8) | 235.0  (4.9, 442.1) | 431.5  (359.2, 491.4) | 54.4  (29.2, 77.2) | 74.2  (66.2, 80.7) |
| 2010 | 544.2  (430.6, 657.8) | 43.2  (26.3, 58.6) | -3.3  (-27.9, 21.9) | 18.7  (15.1, 22.7) | 3.6  (-9.5, 16.0) | 14.5  (12.5, 16.4) | 3.2  (-16.4, 21.9) | 31.4  (28.4, 34.2) | 31.1  (-29.5, 95.2) | 68.7  (57.8, 78.4) | 237.2  (4.9, 446.2) | 219.9  (183.1, 250.4) | 54.9  (29.4, 77.9) | 37.9  (33.9, 41.2) |
| 2011 | 572.2  (452.8, 691.7) | 71.9  (43.7, 97.5) | -3.5  (-29.0, 22.8) | 30.8  (24.9, 37.3) | 3.7  (-9.9, 16.7) | 23.8  (20.6, 26.9) | 3.3  (-17.0, 22.8) | 51.4  (46.4, 56.0) | 32.6  (-30.9, 99.7) | 113.4  (95.4, 129.3) | 246.6  (5.1, 464.1) | 360.4  (300.0, 410.4) | 57.3  (30.6, 81.5) | 62.7  (55.9, 68.1) |
| 2012 | 594.7  (470.6, 718.9) | 97.6  (59.4, 132.4) | -3.6  (-30.3, 23.8) | 42.0  (33.9, 50.9) | 3.9  (-10.4, 17.4) | 32.4  (28.0, 36.7) | 3.5  (-17.7, 23.8) | 70.2  (63.4, 76.5) | 33.8  (-32.1, 103.4) | 153.6  (129.2, 175.2) | 257.1  (5.3, 483.8) | 491.4  (409.1, 559.6) | 60.0  (32.1, 85.4) | 85.8  (76.5, 93.3) |
| 2013 | 531.2  (420.3, 642.1) | 57.8  (35.2, 78.5) | -3.3  (-27.2, 21.3) | 25.0  (20.2, 30.3) | 3.5  (-9.3, 15.6) | 19.3  (16.6, 21.8) | 3.1  (-15.9, 21.3) | 41.8  (37.7, 45.5) | 30.2  (-28.7, 92.4) | 91.2  (76.7, 104.0) | 230.8  (4.8, 434.2) | 293.1  (244.0, 333.8) | 53.9  (28.8, 76.8) | 51.3  (45.7, 55.7) |
| 2014 | 581.7  (460.3, 703.1) | 101.4  (61.7, 137.6) | -3.6  (-29.8, 23.4) | 44.0  (35.5, 53.3) | 3.8  (-10.2, 17.1) | 33.8  (29.2, 38.3) | 3.4  (-17.4, 23.4) | 73.4  (66.3, 80.0) | 33.1  (-31.4, 101.3) | 160.1  (134.7, 182.7) | 253.0  (5.2, 476.1) | 514.7  (428.5, 586.1) | 59.2  (31.4, 84.4) | 90.6  (80.7, 98.5) |
| 2015 | 630.6  (499.0, 762.2) | 111.5  (67.8, 151.3) | -3.9  (-32.2, 25.3) | 48.2  (39.0, 58.4) | 4.1  (-11.0, 18.4) | 37.1  (32.0, 41.9) | 3.7  (-18.9, 25.3) | 80.6  (72.8, 87.8) | 35.7  (-34.0, 109.5) | 175.5  (147.6, 200.2) | 273.8  (5.7, 515.1) | 564.7  (470.0, 643.0) | 64.0  (33.9, 91.6) | 99.9  (89.0, 108.6) |
| 2016 | 663.6  (525.1, 802.1) | 97.2  (59.1, 131.9) | -4.1  (-34.1, 26.8) | 42.4  (34.2, 51.3) | 4.3  (-11.6, 19.5) | 32.6  (28.2, 36.9) | 3.9  (-20.0, 26.8) | 70.7  (63.8, 77.0) | 37.7  (-35.9, 115.6) | 153.3  (129.0, 174.9) | 290.2  (6.0, 546.1) | 496.4  (413.2, 565.2) | 67.7  (35.6, 97.1) | 88.2  (78.6, 95.9) |
| 2017 | 646.6  (511.7, 781.6) | 180.5  (109.8, 244.9) | -4.0  (-33.4, 26.2) | 79.0  (63.8, 95.7) | 4.2  (-11.4, 19.1) | 60.8  (52.5, 68.8) | 3.8  (-19.5, 26.1) | 131.6  (118.8, 143.4) | 36.9  (-35.1, 113.1) | 286.4  (240.9, 326.7) | 283.6  (5.9, 533.6) | 925.9  (770.7, 1054.3) | 65.8  (34.3, 94.8) | 165.4  (147.4, 179.8) |
| **Average**^†^ | **589.8**  **(466.8, 719.9)** | **95.1**  **(57.2, 129.1)** | **-3.6**  **(-30.2, 24.0)** | **41.3**  **(32.9, 50.0)** | **3.8**  **(-10.3, 17.5)** | **31.8**  **(27.1, 36.0)** | **3.5**  **(-17.7, 23.9)** | **68.9**  **(61.5, 75.0)** | **33.5**  **(-31.9, 103.8)** | **150.3**  **(125.0, 171.4)** | **256.4**  **(5.3, 487.4)** | **483.3**  **(397.5, 550.4)** | **59.7**  **(31.7, 86.2)** | **85.2**  **(147.4, 179.8)** |
| Notes:  ^†^Calculation for the annual average seasonal influenza-attributable hospitalisation excluded the pandemic year 2009.  ^‡^Calculation for the all ages estimates included both significant and non-significant values. | | | | | | | | | | | | | | |

**Supplementary Table 3. Estimated annual and average rate (95% confidence interval) per 100,000 population of pneumonia and influenza (P&I) hospitalisations attributable to RSV and influenza, Australia, 2009-2017**

|  | **Age 0 to 4 years** | | **Age 5 to 14 years** | | **Age 15 to 44 years** | | **Age 45 to 64 years** | | **Age 65 to 74 years** | | **Age ≥75 years** | | **All ages**^‡^ | |
| --- | --- | --- | --- | --- | --- | --- | --- | --- | --- | --- | --- | --- | --- | --- |
| **Year** | **RSV** | **Influenza** | **RSV** | **Influenza** | **RSV** | **Influenza** | **RSV** | **Influenza** | **RSV** | **Influenza** | **RSV** | **Influenza** | **RSV** | **Influenza** |
| 2009 | 80.5  (41.9, 116.1) | 80.6  (69.5, 90.5) | 4.0  (-11.6, 19.5) | 26.3  (22.0, 30.9) | 2.7  (-6.3, 11.4) | 23.2  (20.5, 25.8) | -6.3  (-18.0, 5.3) | 41.2  (37.7, 44.2) | -4.5  (-41.3, 11.7) | 79.8  (67.6, 89.9) | 117.3  (-10.4, 264.7) | 291.3  (244.1, 334.8) | 12.3  (-1.9, 26.0) | 52.5  (47.7, 56.3) |
| 2010 | 80.5  (42.0, 116.1) | 40.6  (35.0, 45.5) | 4.0  (-11.7, 19.7) | 13.4  (11.2, 15.8) | 2.8  (-6.3, 11.5) | 11.8  (10.4, 13.1) | -6.3  (-18.1, 5.3) | 20.9  (19.1, 22.4) | -4.5  (-41.6, 30.0) | 40.7  (34.5, 45.8) | 118.4  (-26.9, 267.2) | 148.4  (124.4, 170.6) | 12.5  (-1.9, 26.3) | 26.8  (24.3, 28.7) |
| 2011 | 84.6  (44.1, 122.1) | 67.5  (58.2, 75.7) | 4.2  (-12.2, 20.5) | 22.0  (18.5, 25.9) | 2.9  (-6.6, 12.0) | 19.4  (17.1, 21.6) | -6.5  (-18.8, 5.6) | 34.2  (31.3, 36.7) | -4.7  (-43.6, 30.3) | 67.1  (56.8, 75.6) | 123.2  (-27.1, 277.9) | 243.3  (203.8, 279.6) | 13.0  (-2.0, 27.6) | 44.2  (40.2, 47.4) |
| 2012 | 88.0  (45.9, 126.9) | 91.7  (79.1, 102.9) | 4.4  (-12.8, 21.4) | 30.1  (25.2, 35.4) | 3.0  (-6.8, 12.5) | 26.4  (23.3, 29.3) | -6.8  (-19.6, 5.8) | 46.7  (42.8, 50.1) | -4.9  (-45.2, 31.3) | 90.9  (77.0, 102.4) | 128.4  (-28.2, 289.7) | 331.7  (277.9, 381.2) | 13.6  (-2.1, 28.8) | 60.5  (55.0, 64.9) |
| 2013 | 78.6  (41.0, 113.4) | 54.3  (46.9, 60.9) | 3.9  (-11.4, 19.2) | 17.9  (15.0, 21.1) | 2.7  (-6.1, 11.2) | 15.7  (13.9, 17.4) | -6.1  (-17.6, 5.2) | 27.8  (25.5, 29.8) | -4.4  (-40.4, 32.7) | 54.0  (45.7, 60.8) | 115.3  (-29.4, 260.0) | 197.9  (165.8, 227.4) | 12.3  (-1.9, 26.0) | 36.2  (32.8, 38.8) |
| 2014 | 86.0  (44.8, 124.2) | 95.3  (82.2, 106.9) | 4.3  (-12.5, 21.1) | 31.5  (26.4, 37.1) | 3.0  (-6.7, 12.3) | 27.5  (24.4, 30.6) | -6.7  (-19.3, 5.7) | 48.8  (44.7, 52.4) | -4.8  (-44.2, 29.5) | 94.8  (80.3, 106.7) | 126.4  (-26.3, 285.1) | 347.4  (291.1, 399.3) | 13.5  (-2.2, 28.7) | 63.8  (57.9, 68.5) |
| 2015 | 93.3  (48.6, 134.6) | 104.7  (90.3, 117.5) | 4.6  (-13.6, 22.8) | 34.5  (28.9, 40.6) | 3.2  (-7.3, 13.3) | 30.2  (26.7, 33.5) | -7.3  ( -20.9, 6.2) | 53.6  (49.1, 57.5) | -5.2  (-47.8, 32.3) | 103.9  (88.0, 117.0) | 136.7  (-28.9, 308.4) | 381.1  (319.4, 438.1) | 14.6  (-2.4, 31.2) | 70.3  (63.8, 75.5) |
| 2016 | 98.1  (51.2, 141.6) | 91.3  (78.7, 102.4) | 4.9  (-14.4, 24.2) | 30.3  (25.4, 35.7) | 3.4  (-7.7, 14.1) | 26.5  (23.4, 29.5) | -7.7  (-22.1, 6.5) | 47.0  (43.1, 50.5) | -5.5  (-50.5, 34.9) | 90.7  (76.9, 102.2) | 145.0  (-31.2, 327.0) | 335.1  (280.7, 385.1) | 15.6  (-2.7, 33.3) | 62.0  (56.2, 66.6) |
| 2017 | 95.6  (49.9, 138.0) | 169.6  (146.3, 190.2) | 4.8  (-14.0, 23.6) | 56.5  (47.4, 66.6) | 3.3  (-7.5, 13.8) | 49.5  (43.7, 55.0) | -7.5  (-21.6, 6.4) | 87.5  (80.2, 93.9) | -5.3  (-49.4, 37.1) | 169.5  (143.6, 190.9) | 141.6  (-32.9, 319.5) | 624.9  (523.6, 718.3) | 15.2  (-2.8, 32.7) | 116.2  (105.3, 124.9) |
| **Average**^†^ | **87.2**  **(45.5, 127.1)** | **89.4**  **(76.2, 100.3)** | **4.3**  **(-12.7, 21.6)** | **29.5**  **(24.5, 34.8)** | **3.0**  **(-6.8, 12.6)** | **25.9**  **(22.6, 28.8)** | **-6.8**  **(-19.6, 5.8)** | **45.8**  **(41.5, 49.2)** | **-4.9**  **(-44.9, 32.3)** | **88.9**  **(74.5, 100.2)** | **128.0**  **(-26.8, 291.9)** | **326.2**  **(270.1, 375.0)** | **13.6**  **(-2.2, 29.3)** | **60.0**  **(53.7, 64.4)** |
| Notes:  ^†^Calculation for the annual average seasonal influenza-attributable hospitalisation excluded the pandemic year 2009.  ^‡^Calculation for the all ages estimates included both significant and non-significant values. | | | | | | | | | | | | | | |

**Supplementary Table 4. Estimated annual and average rate (95% confidence interval) per 100,000 population of bronchiolitis hospitalisations attributable to RSV and influenza, Australia, 2009-2017**

|  | **Age 0 to 4 years** | |  |  |  |  |  |  |  |  |  |  |  |  |  |  |  |  |
| --- | --- | --- | --- | --- | --- | --- | --- | --- | --- | --- | --- | --- | --- | --- | --- | --- | --- | --- |
| **Year** | **RSV** | **Influenza** |  |  |  |  |  |  |  |  |  |  |  |  |  |  |  |  |
| 2009 | 430.6  (385.6, 479.8) | -15.8  (-30.7, -4.2) |  |  |  |  |  |  |  |  |  |  |  |  |  |  |  |  |
| 2010 | 430.7  (385.8, 480.0) | -7.9  (-15.4, -2.1) |  |  |  |  |  |  |  |  |  |  |  |  |  |  |  |  |
| 2011 | 452.9  (405.6, 504.7) | -13.2  (-25.7, -3.5) |  |  |  |  |  |  |  |  |  |  |  |  |  |  |  |  |
| 2012 | 470.7  (421.6, 524.6) | -17.9  (-34.9, -4.8) |  |  |  |  |  |  |  |  |  |  |  |  |  |  |  |  |
| 2013 | 420.5  (376.6, 468.6) | -10.6  (-20.7, -2.9) |  |  |  |  |  |  |  |  |  |  |  |  |  |  |  |  |
| 2014 | 460.4  (412.4, 513.1) | -18.6  (-36.2, -5.0) |  |  |  |  |  |  |  |  |  |  |  |  |  |  |  |  |
| 2015 | 499.1  (447.0, 556.2) | -20.5  (-39.8, -5.5) |  |  |  |  |  |  |  |  |  |  |  |  |  |  |  |  |
| 2016 | 525.3  (470.4, 585.3) | -17.8  (-34.7, -4.8) |  |  |  |  |  |  |  |  |  |  |  |  |  |  |  |  |
| 2017 | 511.8  (458.4, 570.4) | -33.1  (-64.5, -8.9) |  |  |  |  |  |  |  |  |  |  |  |  |  |  |  |  |
| **Average**^†^ | **466.9**  **(418.2, 525.4)** | **-17.5**  **(-33.6, -4.7)** |  |  |  |  |  |  |  |  |  |  |  |  |  |  |  |  |
| Notes:  ^†^Calculation for the annual average seasonal influenza-attributable hospitalisation excluded the pandemic year 2009. | | | | | | | | | | | | | | | | | | |

**Supplementary Table 5. Estimated annual and average (95% confidence interval) number of respiratory hospitalisations attributable to RSV and influenza, Australia, 2009-2017**

|  | **Age 0 to 4 years** | | **Age 5 to 14 years** | | **Age 15 to 44 years** | | **Age 45 to 64 years** | | **Age 65 to 74 years** | | **Age ≥75 years** | | **All ages**^‡^ | |
| --- | --- | --- | --- | --- | --- | --- | --- | --- | --- | --- | --- | --- | --- | --- |
| **Year** | **RSV** | **Influenza** | **RSV** | **Influenza** | **RSV** | **Influenza** | **RSV** | **Influenza** | **RSV** | **Influenza** | **RSV** | **Influenza** | **RSV** | **Influenza** |
| 2009 | 6103  (3759, 8349) | 740  (68, 1385) | -1806  (-3195, -473) | 639  (255, 1067) | 609  (-1167, 2366) | 2797  (2231, 3345) | 440  (-1092, 1902) | 3808  (3334, 4254) | 915  (-238, 2164) | 2291  (1896, 2643) | 4432  (974, 7652) | 6262  (5114, 7202) | 10693  (3911, 17074) | 16536  (14226, 18463) |
| 2010 | 6227  (3836, 8518) | 380  (35, 710) | -1835  (-3247, -480) | 327  (131, 547) | 621  (-1190, 2413) | 1437  (1146, 1719) | 450  (-1118, 1947) | 1966  (1721, 2196) | 962  (-250, 2276) | 1219  (1008, 1406) | 4570  (1004, 7890) | 3260  (2662, 3749) | 10995  (4027, 17559) | 8588  (7393, 9579) |
| 2011 | 6566  (4044, 8982) | 633  (58, 1186) | -1932  (-3418, -506) | 544  (217, 909) | 655  (-1254, 2542) | 2391  (1907, 2860) | 474  (-1177, 2050) | 3263  (2857, 3645) | 1051  (-273, 2486) | 2096  (1734, 2418) | 4865  (1069, 8400) | 5468  (4466, 6290) | 11678  (4265, 18660) | 14396  (12399, 16046) |
| 2012 | 6995  (4309, 9570) | 882  (81, 1651) | -2041  (-3611, -534) | 752  (300, 1256) | 692  (-1326, 2689) | 3307  (2638, 3956) | 499  (-1238, 2157) | 4492  (3933, 5018) | 1151  (-299, 2722) | 3000  (2482, 3459) | 5185  (1139, 8952) | 7623  (6226, 8768) | 12481  (4592, 19922) | 20056  (17280, 22343) |
| 2013 | 6372  (3925, 8718) | 533  (49, 998) | -1859  (-3289, -486) | 455  (182, 759) | 629  (-1205, 2443) | 1993  (1590, 2384) | 452  (-1124, 1958) | 2706  (2369, 3023) | 1076  (-280, 2545) | 1864  (1542, 2150) | 4764  (1047, 8225) | 4655  (3801, 5354) | 11435  (4226, 18261) | 12204  (10520, 13586) |
| 2014 | 7056  (4346, 9653) | 945  (87, 1769) | -2072  (-3666, -542) | 812  (325, 1357) | 697  (-1335, 2707) | 3539  (2823, 4234) | 502  ( -1248, 2173) | 4813  (4214, 5377) | 1226  (-319, 2900) | 3401  (2814, 3923) | 5359  (1178, 9253) | 8387  (6850, 9647) | 12769  (4730, 20428) | 21897  (18881, 24363) |
| 2015 | 7704  (4746, 10540) | 1047  (97, 1959) | -2279  (-4031, -596) | 905  (362, 1512) | 761  (-1458, 2956) | 3916  (3124, 4685) | 550  (-1366, 2380) | 5343  (4678, 5969) | 1377  (-358, 3256) | 3871  (3203, 4465) | 5944  (1306, 10262) | 9431  (7703, 10848) | 14058  (5203, 22535) | 24514  (21141, 27264) |
| 2016 | 8218  (5062, 11242) | 925  (85, 1731) | -2455  (-4344, -642) | 809  (323, 1351) | 814  (-1560, 3163) | 3476  ( 2773, 4158) | 590  (-1466, 2554) | 4754  (4162, 5311) | 1509  (-392, 3568) | 3511  (2905, 4049) | 6470  (1422, 11171) | 8513  (6952, 9791) | 15146  (5549, 24350) | 21987  (18963, 24450) |
| 2017 | 8035  (4949, 10992) | 1723  ( 159, 3226) | -2449  (-4333, -641) | 1540  (615, 2573) | 807  (-1546, 3135) | 6576  (5246, 7867) | 585  (-1452, 2530) | 8984  ( 7866, 10036) | 1524  (-396, 3604) | 6772  (5604, 7811) | 6523  (1433, 11262) | 16380  (13378, 18841) | 15024  (5417, 24223) | 41976  (36206, 46663) |
| **Average**^†^ | **7031**  **(4331, 9618)** | **883**  **(82, 1654)** | **-2081**  **(-3682, -544)** | **768**  **(307, 1283)** | **698**  **(-1338, 2713)** | **3330**  **(2656, 3983)** | **505**  **(-1253, 2183)** | **4540**  **(3975, 5072)** | **1199**  **(-312, 2836)** | **3217**  **(2662, 3710)** | **5346**  **(1175, 9230)** | **7965**  **(6505, 9161)** | **12698**  **(4679, 20334)** | **20702**  **(17851, 23025)** |
| Notes:  ^†^Calculation for the annual average seasonal influenza-attributable hospitalisation excluded the pandemic year 2009.  ^‡^Calculation for the all ages estimates included both significant and non-significant values. | | | | | | | | | | | | | | |

**Supplementary Table 6. Estimated annual and average (95% confidence interval) number of acute respiratory infection (ARI) hospitalisations attributable to RSV and influenza, Australia, 2009-2017**

|  | **Age 0 to 4 years** | | **Age 5 to 14 years** | | **Age 15 to 44 years** | | **Age 45 to 64 years** | | **Age 65 to 74 years** | | **Age ≥75 years** | | **All ages**^‡^ | |
| --- | --- | --- | --- | --- | --- | --- | --- | --- | --- | --- | --- | --- | --- | --- |
| **Year** | **RSV** | **Influenza** | **RSV** | **Influenza** | **RSV** | **Influenza** | **RSV** | **Influenza** | **RSV** | **Influenza** | **RSV** | **Influenza** | **RSV** | **Influenza** |
| 2009 | 7755  (6137, 9374) | 1224  (744, 1660) | -90  (-753, 592) | 1003  (810, 1215) | 326  (-872, 1463) | 2624  (2267, 2969) | 173  (-882,1182) | 3358  (3033, 3659) | 477  (-453, 1461) | 2085  (1753, 2378) | 3159  (65,5944) | 5802  (4829, 6607) | 11799  (6326, 16737) | 16096  (14370, 17511) |
| 2010 | 7912  (6261, 9564) | 628  (382, 852) | -92  (-765, 601) | 514  (415, 623) | 332  (-889, 1492) | 1348  (1165, 1525) | 177  (-903,1211) | 1734  (1566, 1889) | 501  (-477, 1536) | 1109  (933, 1265) | 3257  (67,6128) | 3020  (2514, 3439) | 12088  (6469, 17168) | 8353  (7459, 9085) |
| 2011 | 8343  (6602, 10085) | 1048  (637, 1422) | -97  (-806, 633) | 854  (690, 1035) | 350  (-937, 1572) | 2243  (1939, 2538) | 187  (-951,1274) | 2878  (2599, 3136) | 548  (-520, 1678) | 1907  (1604, 2175) | 3468  (72,6525) | 5067  (4218, 5770) | 12798  (6838, 18202) | 13997  (12499, 15221) |
| 2012 | 8889  (7034, 10745) | 1459  (887, 1980) | -102  (-851, 669) | 1181  (953, 1430) | 370  (-991, 1663) | 3103  (2681, 3511) | 197  (-1001,1341) | 3962  (3578, 4317) | 600  (-570, 1837) | 2729  (2296, 3113) | 3696  (76,6954) | 7063  (5880, 8043) | 13648  (7307, 19410) | 19498  (17401, 21200) |
| 2013 | 8097  (6408, 9788) | 881  (536, 1196) | -93  (-775, 609) | 714  (577, 865) | 336  (-900, 1510) | 1870  (1616, 2116) | 178  (-908,1217) | 2386  (2155, 2600) | 561  (-533, 1718) | 1696  (1426, 1934) | 3396  (70,6389) | 4313  (3590, 4911) | 12475  (6663, 17762) | 11860  (10580, 12893) |
| 2014 | 8966  (7095, 10838) | 1563  (951, 2121) | -104  (-864, 679) | 1275  (1030, 1545) | 372  (-998, 1674) | 3321  (2869, 3757) | 198  (-1008,1351) | 4245  (3834, 4625) | 639  (-607, 1957) | 3095  (2603, 3530) | 3820  (79,7187) | 7771  (6469, 8849) | 13892  (7377, 19822) | 21270  (18953, 23119) |
| 2015 | 9790  (7747, 11834) | 1731  (1053, 2349) | -114  (-950, 747) | 1422  (1148, 1722) | 407  (-1089, 1828) | 3674  (3175, 4157) | 217  (-1104,1480) | 4713  (4256, 5135) | 717  (-682, 2198) | 3522  (2963, 4018) | 4237  (88,7971) | 8739  (7274, 9951) | 15253  (8067, 21813) | 23801  (21206, 25869) |
| 2016 | 10442  (8263, 12622) | 1529  (930, 2075) | -123  (-1024, 804) | 1271  (1026, 1539) | 435  (-1165, 1955) | 3262  (2818, 3690) | 233  (-1185,1588) | 4193  (3787, 4569) | 786  (-747, 2408) | 3194  (2687, 3644) | 4612  (95,8677) | 7887  (6566, 8982) | 16385  (8621, 23496) | 21336  (19007, 23190) |
| 2017 | 10210  (8079, 12342) | 2850  (1733, 3867) | -123  (-1021, 802) | 2419  (1953, 2930) | 431  (-1155, 1938) | 6170  (5332, 6981) | 231  (-1174,1573) | 7924  (7157, 8634) | 794  (-755, 2433) | 6162  (5183, 7028) | 4649  (96,8748) | 15177  (12634, 17283) | 16192  (8440, 23317) | 40702  (36251, 44235) |
| **Average**^†^ | **8934**  **(7069, 10799)** | **1461**  **(889, 1982)** | **-104**  **(-868, 682)** | **1206**  **(974, 1461)** | **373**  **(-1000, 1677)** | **3124**  **(2699, 3535)** | **199**  **(-1013, 1357)** | **4004**  **(3617, 4363)** | **625**  **(-594, 1914)** | **2927**  **(2462, 3338)** | **3810**  **(79, 7169)** | **7380**  **(6143, 8404)** | **13837**  **(7345, 19747)** | **20102**  **(17909, 21849)** |
| Notes:  ^†^Calculation for the annual average seasonal influenza-attributable hospitalisation excluded the pandemic year 2009.  ^‡^Calculation for the all ages estimates included both significant and non-significant values. | | | | | | | | | | | | | | |

**Supplementary Table 7. Estimated annual and average (95% confidence interval) number of pneumonia and influenza (P&I) hospitalisations attributable to RSV and influenza, Australia, 2009-2017**

|  | **Age 0 to 4 years** | | **Age 5 to 14 years** | | **Age 15 to 44 years** | | **Age 45 to 64 years** | | **Age 65 to 74 years** | | **Age ≥75 years** | | **All ages**^‡^ | |
| --- | --- | --- | --- | --- | --- | --- | --- | --- | --- | --- | --- | --- | --- | --- |
| **Year** | **RSV** | **Influenza** | **RSV** | **Influenza** | **RSV** | **Influenza** | **RSV** | **Influenza** | **RSV** | **Influenza** | **RSV** | **Influenza** | **RSV** | **Influenza** |
| 2009 | 1147  (598, 1655) | 1149  (991, 1290) | 108  (-317, 533) | 717  (602, 845) | 253  (-577, 1054) | 2136  (1888, 2375) | -339  (-977, 288) | 2234  (2047, 2398) | -69  (-638, 484) | 1234  (1045, 1390) | 1578  (-370, 3559) | 3916  (3281, 4501) | 2678  (-402, 5642) | 11387  (10346, 12208) |
| 2010 | 1170  (610, 1689) | 590  (509, 662) | 110  (-322, 542) | 368  (308, 433) | 258  (-588, 1074) | 1097  (970, 1220) | -348  (-1001, 295) | 1153  (1057, 1238) | -73  (-671, 509) | 656  (556, 739) | 1627  (-381, 3670) | 2039  (1708, 2343) | 2745  (-416, 5801) | 5903  (5363, 6328) |
| 2011 | 1234  (643, 1781) | 984  (849, 1104) | 116  (-339, 570) | 611  (513, 720) | 272  (-620, 1132) | 1826  (1614, 2030) | -366  (-1054, 311) | 1914  (1754, 2055) | -79  (-733, 556) | 1129  (956, 1271) | 1732  (-406, 3907) | 3420  (2866, 3931) | 2909  (-444, 6160) | 9884  (8978, 10594) |
| 2012 | 1315  (685, 1897) | 1371  (1182, 1538) | 123  (-358, 602) | 844  (708, 995) | 288  (-655, 1198) | 2526  (2233, 2808) | -385  (-1109, 327) | 2636  (2415, 2829) | -87  (-803, 609) | 1615  (1368, 1819) | 1846  (-433, 4164) | 4768  (3995, 5480) | 3099  (-477, 6550) | 13759  (12495, 14746) |
| 2013 | 1198  (624, 1728) | 828  (714, 929) | 112  (-326, 549) | 511  (428, 602) | 262  (-595, 1088) | 1522  (1345, 1692) | -349  (-1006, 297) | 1588  (1455, 1704) | -81  (-750, 569) | 1004  (850, 1130) | 1696  (-397, 3826) | 2911  (2439, 3346) | 2836  (-443, 6010) | 8363  (7592, 8966) |
| 2014 | 1326  (691, 1914) | 1468  (1266, 1647) | 124  (-364, 612) | 912  (765, 1074) | 290  (-660, 1206) | 2703  (2389, 3005) | -388  (-1117, 329) | 2824  (2587, 3031) | -93  (-855, 649) | 1831  (1551, 2063) | 1908  (-447, 4304) | 5245  (4395, 6029) | 3168  (-508, 6737) | 14984  (13599, 16073) |
| 2015 | 1448  (755, 2090) | 1626  (1403, 1824) | 137  (-400, 672) | 1016  (853, 1198) | 317  (-721, 1317) | 2991  (2644, 3325) | -425  (-1223, 361) | 3135  (2872, 3365) | -104  (-960, 728) | 2084  (1766, 2348) | 2116  (-496, 4773) | 5899  (4942, 6780) | 3488  (-579, 7441) | 16752  (15198, 17979) |
| 2016 | 1544  (805, 2229) | 1437  (1239, 1612) | 147  (-431, 725) | 908  (762, 1070) | 339  (-771, 1408) | 2655  (2347, 2952) | -456  (-1313, 387) | 2789  (2556, 2994) | -114  (-1052, 798) | 1890  (1601, 2129) | 2303  (-540, 5196) | 5324  (4461, 6119) | 3764  (-649, 8056) | 15004  (13603, 16112) |
| 2017 | 1510  (787, 2179) | 2677  (2309, 3004) | 147  (-430, 723) | 1729  (1451, 2038) | 336  (-764, 1396) | 5023  (4440, 5584) | -452  (-1300, 384) | 5271  (4829, 5658) | -115  (-1063, 806) | 3647  (3089, 4107) | 2322  (-544, 5238) | 10245  (8584, 11775) | 3748  (-680, 8054) | 28592  (25909, 30727) |
| **Average**^†^ | **1321**  **(689, 1907)** | **1373**  **(1184, 1540)** | **125**  **(-365, 614)** | **862**  **(724, 1016)** | **290**  **(-661, 1208)** | **2543**  **(2248, 2827)** | **-390**  **(-1122, 331)** | **2664**  **(2441, 2859)** | **-91**  **(-836, 634)** | **1732**  **(1467, 1951)** | **1903**  **(-446, 4293)** | **4981**  **(4174, 5725)** | **3159**  **(-501, 6720)** | **14155**  **(12842, 15189)** |
| Notes:  ^†^Calculation for the annual average seasonal influenza-attributable hospitalisation excluded the pandemic year 2009.  ^‡^Calculation for the all ages estimates included both significant and non-significant values. | | | | | | | | | | | | | | |

**Supplementary Table 8. Estimated annual and average (95% confidence interval) number of bronchiolitis hospitalisations attributable to RSV and influenza, Australia, 2009-2017**

|  | **Age 0 to 4 years** | |  |  |  |  |  |  |  |  |  |  |  |  |  |  |  |  |
| --- | --- | --- | --- | --- | --- | --- | --- | --- | --- | --- | --- | --- | --- | --- | --- | --- | --- | --- |
| **Year** | **RSV** | **Influenza** |  |  |  |  |  |  |  |  |  |  |  |  |  |  |  |  |
| 2009 | 6139  (5498, 6841) | -225  (-437, -60) |  |  |  |  |  |  |  |  |  |  |  |  |  |  |  |  |
| 2010 | 6263  (5609, 6979) | -115  (-224, -31) |  |  |  |  |  |  |  |  |  |  |  |  |  |  |  |  |
| 2011 | 6604  (5915, 7359) | -192  (-374, -52) |  |  |  |  |  |  |  |  |  |  |  |  |  |  |  |  |
| 2012 | 7036  (6302, 7841) | -268  (-521, -72) |  |  |  |  |  |  |  |  |  |  |  |  |  |  |  |  |
| 2013 | 6410  (5741, 7143) | -162  (-315, -43) |  |  |  |  |  |  |  |  |  |  |  |  |  |  |  |  |
| 2014 | 7097  (6357, 7909) | -287  (-558, -77) |  |  |  |  |  |  |  |  |  |  |  |  |  |  |  |  |
| 2015 | 7749  (6941, 8636) | -318  (-619, -85) |  |  |  |  |  |  |  |  |  |  |  |  |  |  |  |  |
| 2016 | 8266  (7403, 9211) | -281  (-546, -75) |  |  |  |  |  |  |  |  |  |  |  |  |  |  |  |  |
| 2017 | 8082  (7238, 9006) | -523  (-1018, -141) |  |  |  |  |  |  |  |  |  |  |  |  |  |  |  |  |
| **Average**^†^ | **7017**  **(6285, 7820)** | **-255**  **(-497, -69)** |  |  |  |  |  |  |  |  |  |  |  |  |  |  |  |  |
| Notes:  ^†^Calculation for the annual average seasonal influenza-attributable hospitalisation excluded the pandemic year 2009. | | | | | | | | | | | | | | | | | | |

**Supplementary Table 9. Estimated age-standardised all-age rates of hospitalisation (95% confidence interval) attributable to RSV and influenza, by diagnostic category and year, Australia, 2009-2017**

|  | **P&I** | | **ARI** | | **Respiratory** | |
| --- | --- | --- | --- | --- | --- | --- |
| **Year** | **RSV** | **Influenza** | **RSV** | **Influenza** | **RSV** | **Influenza** |
| 2009 | 12.7  (-10.2, 34.7) | 54.7  (47.3, 61.6) | 54.7  (13.5, 94.2) | 78.0  (13.5, 94.2) | 50.9  (-4.6, 104.7) | 80.5  (63.0, 96.5) |
| 2010 | 12.8  (-11.4, 36.6) | 27.8  (24.0, 31.3) | 54.9  (13.4, 94.7) | 39.7  (13.4, 94.7) | 51.1  ( -4.8, 105.3) | 40.9  (32.1, 49.1) |
| 2011 | 13.4  (-11.8, 38.0) | 45.7  (39.5, 51.5) | 57.5  (14.2, 99.0) | 65.2  (14.2, 99.0) | 53.5  (-4.8, 110.1) | 67.2  (52.7, 80.6) |
| 2012 | 13.9  (-12.2, 39.6) | 62.3  (53.8, 70.1) | 59.8  (14.8, 103.1) | 88.7  (14.8, 103.1) | 55.7  (-5.1, 114.6) | 91.6  (71.7, 109.8) |
| 2013 | 12.5  (-11.3, 35.9) | 37.1  (32.0, 41.7) | 53.5  (13.1, 92.3) | 52.8  (13.1, 92.3) | 49.8  (-4.6, 102.6) | 54.5  (42.7, 65.3) |
| 2014 | 13.6  (-11.9, 38.8) | 65.1  (56.2, 73.2) | 58.6  (14.4, 101.1) | 92.8  (14.4, 101.1) | 54.6  (-5.1, 112.4) | 95.7  (75.0, 114.8) |
| 2015 | 14.8  (-12.9, 42.0) | 71.4  (61.7, 80.3) | 63.5  (15.6, 109.5) | 101.7  (15.6, 109.5) | 59.1  (-5.5, 121.7) | 105.0  (82.3, 125.9) |
| 2016 | 15.6  (-13.7, 44.5) | 62.6  (54.1, 70.5) | 67.0  (16.3, 115.6) | 89.3  (16.3, 115.6) | 62.4  (-5.9, 128.6) | 92.1  (72.2, 110.4) |
| 2017 | 15.3  (-13.6, 43.8) | 116.8  (100.9, 131.4) | 65.3  (15.9, 112.8) | 166.4  (15.9, 112.8) | 60.9  (-5.8, 125.5) | 171.8  (134.6, 205.9) |
| **Average**^†^ | **13.8**  **(-12.1, 39.3)** | **60.4**  **(52.2, 68.0)** | **59.4**  **(14.6, 103.5)** | **87.1**  **(14.6, 103.5)** | **55.3**  **(-5.1, 115.1)** | **89.9**  **(69.6, 107.7)** |
| Notes:  ^†^Calculation for the annual average seasonal influenza-attributable hospitalisation excluded the pandemic year 2009.  ARI, acute respiratory infection  P&I, pneumonia and influenza | | | | | | |

**Supplementary Table 10. Summary statistics for the weekly number of hospitalisations (principal diagnosis) for each diagnostic category, Australia, 1 July 2008-30 June 2018^†^**

|  |  | **Basic statistical measures** | | | | | | | | |
| --- | --- | --- | --- | --- | --- | --- | --- | --- | --- | --- |
| **Diagnostic category** |  | **Mean** | **Median** | **Mode** | **Standard**  **deviation** | **Variance** | **Minimum** | **Maximum** | **Range** | **IQR** |
| Respiratory | All-age | 5549.1 | 5420.5 | 3931 | 1320 | 1743313 | 3112 | 10308 | 7196 | 1724 |
|  | 0-4 | 1163.0 | 1188.5 | 942 | 331.4 | 109809 | 400 | 1911 | 1511 | 491 |
|  | 5-14 | 331.9 | 337 | 280 | 90.5 | 8195 | 104 | 654 | 550 | 111 |
|  | 15-44 | 810.4 | 781.5 | 691 | 162.3 | 26335 | 527 | 1340 | 813 | 211.5 |
|  | 45-64 | 882.5 | 845.5 | 661 | 225.3 | 50743 | 501 | 1787 | 1286 | 275.5 |
|  | 65-74 | 789.8 | 754.5 | 635 | 214.1 | 45854 | 416 | 1673 | 1257 | 235 |
|  | ≥75 | 1571.5 | 1483 | 1271 | 465.7 | 216909 | 863 | 3576 | 2713 | 492.5 |
| ARI | All-age | 3709.3 | 3520.5 | 2481 | 1109 | 1228994 | 1931 | 8044 | 6113 | 1393 |
|  | 0-4 | 871.0 | 859 | 792 | 296.8 | 88103 | 307 | 1581 | 1274 | 458.5 |
|  | 5-14 | 154.8 | 144 | 150 | 51.9 | 2690 | 48 | 364 | 316 | 55.5 |
|  | 15-44 | 490.8 | 462 | 426 | 124 | 15374 | 297 | 931 | 634 | 140 |
|  | 45-64 | 558.4 | 519 | 496 | 176.9 | 31278 | 276 | 1334 | 1058 | 209 |
|  | 65-74 | 522.7 | 491 | 474 | 170.9 | 29221 | 234 | 1291 | 1057 | 175.5 |
|  | ≥75 | 1111.6 | 1034.5 | 1125 | 391.7 | 153438 | 551 | 2904 | 2353 | 402 |
|  | 0-4 | 344.1 | 323.5 | 211 | 158.6 | 25153 | 66 | 749 | 683 | 245.5 |
| P & I | All-age | 1525.4 | 1408 | 979 | 576.5 | 332321 | 83 | 4425 | 3680 | 583.5 |
|  | 0-4 | 160.0 | 147 | 125 | 70.2 | 4927 | 42 | 403 | 361 | 105.5 |
|  | 5-14 | 66.3 | 58 | 50 | 30.9 | 954.5 | 18 | 212 | 194 | 30.5 |
|  | 15-44 | 205.6 | 180 | 148 | 85.9 | 7385 | 98 | 602 | 504 | 83 |
|  | 45-64 | 277.0 | 254 | 207 | 101.1 | 10211 | 135 | 787 | 652 | 94.5 |
|  | 65-74 | 232.8 | 214 | 184 | 91.1 | 8294 | 95 | 711 | 616 | 88 |
|  | ≥75 | 583.8 | 535 | 562 | 236.5 | 55955 | 275 | 1831 | 1556 | 198.5 |
| Bronchiolitis | All-age | 347.5 | 327 | 197 | 159.5 | 25441 | 66 | 760 | 694 | 248 |
| Notes:  ^†^Principal diagnosis hospital admissions based on the ICD-10-AM codes for all respiratory conditions (ICD J00-J99), acute respiratory infections (ARI; ICD J00-J22 and J44.0), pneumonia and influenza (P&I; ICD J09-J18) or bronchiolitis (ICD J21), as applicable. Hospital admissions that are elective, with length of stay of more than 30 days, and beyond 24 June 2018 were excluded. Please see methods section of main manuscript for further details.  IQR, Interquartile range | | | | | | | | | | |

**Supplementary Table 11. RSV and influenza-coded respiratory hospitalisations (any diagnosis field), Australia, 2009-2017^†^**

|  | **Age 0 to 4 years** | | **Age 5 to 14 years** | | **Age 15 to 44 years** | | **Age 45 to 64 years** | | **Age 65 to 74 years** | | **Age ≥75 years** | | | **All ages** | |
| --- | --- | --- | --- | --- | --- | --- | --- | --- | --- | --- | --- | --- | --- | --- | --- |
| **Year** | **RSV** | **Influenza** | **RSV** | **Influenza** | **RSV** | **Influenza** | **RSV** | **Influenza** | **RSV** | **Influenza** | **RSV** | **Influenza** | **RSV** | | **Influenza** |
| 2009 | 6410 | 1273 | 126 | 656 | 55 | 1766 | 85 | 1432 | 62 | 300 | 110 | 302 | 6838 | | 5613 |
| 2010 | 6540 | 653 | 105 | 186 | 57 | 533 | 105 | 421 | 65 | 102 | 121 | 122 | 6982 | | 1987 |
| 2011 | 6896 | 1090 | 103 | 336 | 70 | 785 | 143 | 634 | 94 | 289 | 137 | 375 | 7437 | | 3477 |
| 2012 | 7347 | 1518 | 163 | 474 | 80 | 1136 | 177 | 1019 | 149 | 795 | 282 | 1621 | 8191 | | 6530 |
| 2013 | 6693 | 917 | 144 | 300 | 127 | 944 | 270 | 1097 | 220 | 554 | 391 | 730 | 7833 | | 4477 |
| 2014 | 7411 | 1626 | 116 | 453 | 141 | 1957 | 339 | 2408 | 322 | 1475 | 599 | 2590 | 8917 | | 10385 |
| 2015 | 8092 | 1801 | 202 | 827 | 272 | 2024 | 566 | 2014 | 555 | 1723 | 1126 | 3452 | 10778 | | 11722 |
| 2016 | 8631 | 1591 | 238 | 507 | 296 | 1961 | 700 | 2496 | 659 | 2130 | 1262 | 4813 | 11743 | | 13333 |
| 2017 | 8439 | 2965 | 260 | 1496 | 361 | 3899 | 884 | 5774 | 1006 | 5284 | 2043 | 12245 | 12944 | | 31361 |
| **Average^‡^** | **7384** | **1520** | **162** | **572** | **162** | **1655** | **363** | **1983** | **348** | **1544** | **675** | **3244** | **9074** | | **10409** |
| Notes:  ^†^Principal or other diagnosis field (any diagnosis field) hospital admissions based on the ICD-10-AM codes for RSV-specific infection (ICD J12.1, J20.5, J21.0, B97.4) or influenza-specific infection (ICD J09, J10.0, J10.1, J10.8), as applicable. Hospital admissions that are elective, with length of stay of more than 30 days, and beyond 24 June 2018 were excluded. Please see methods section of main manuscript for further details.  ^‡^Calculation for the annual average seasonal influenza-coded hospitalisation excluded the pandemic year 2009. | | | | | | | | | | | | | | | |

**Supplementary Table 12. Comparison of selected studies of RSV- and influenza-attributable respiratory hospitalisations (rate per 100,000 population) from the United Kingdom (UK) and United States (US)**

| **Study, country, period** | **Age 0 to 4 years** | | **Age 5 to 14 years** | | **Age 15 to 44 years** | | **Age 45 to 64 years** | | **Age 65 to 74 years** | | **Age ≥75 years** | |
| --- | --- | --- | --- | --- | --- | --- | --- | --- | --- | --- | --- | --- |
|  | **RSV** | **Influenza** | **RSV** | **Influenza** | **RSV** | **Influenza** | **RSV** | **Influenza** | **RSV** | **Influenza** | **RSV** | **Influenza** |
| Nazareno et al., Australia, 2009-2017 | 464  (286, 641) | 58  (5, 108) | -72  (-128, -19) | 26  (10, 44) | 7  (-14, 28) | 34  (27, 41) | 9  (-22, 39) | 78  (68, 87) | 64  (-17, 154) | 165  (135, 191) | 360  (79, 628) | 522  (421, 600) |
| Taylor et al., UK, 1995-2009^4^ | 838  (624, 1011) | -- | 0^†^ | -- | -- | -- | -- | -- | -- | -- | -- | -- |
| Fleming et al., UK, 1995-2009^5^ | -- | -- | -- | -- | 4  (3, 5)^‡^_1_ | -- | 30  (22,36)^‡^_2_ | -- | 86  (62, 101) | -- | 234  (180, 291) | -- |
| Matias et al., US, 1997-2009^6^ | 514  (418, 680) | 128  (52, 173) | 0  (0, 0)^§^_1_ | 20  (12, 33)^§^_1_ | 9  (7, 12)^§^_2_ | 41  (23, 68)^§^_2_ | 28  (22, 36)^§^_3_ | 117  (47, 182)^§^_3_ | 84  (64, 103) | 256  (83, 385) | 258  (201, 307) | 589  (173, 864) |
| Notes:  ^†^5-17 years and statistically significant negative estimates expressed as 0  ^‡^_1_18-49 years; ^‡^_2_50-64 years  ^§^_1_5-17 years; ^§^_2_18-49 years; ^§^_3_50-64 years | | | | | | | | | | | | |

**References**

1. Moa AM, Muscatello DJ, Turner RM, MacIntyre CR. Estimated hospitalisations attributable to seasonal and pandemic influenza in Australia: 2001-2013. *PLoS One*. 2020;15(4):e0230705.

2. Goldstein E, Viboud C, Charu V, Lipsitch M. Improving the estimation of influenza-related mortality over a seasonal baseline. *Epidemiology*. 2012;23(6):829-838.

3. Iuliano AD, Roguski KM, Chang HH, et al. Estimates of global seasonal influenza-associated respiratory mortality: a modelling study. *Lancet*. 2018;391(10127):1285-1300.

4. Taylor S, Taylor RJ, Lustig RL, et al. Modelling estimates of the burden of respiratory syncytial virus infection in children in the UK. *BMJ Open*. 2016;6(6):e009337.

5. Fleming DM, Taylor RJ, Lustig RL, et al. Modelling estimates of the burden of respiratory syncytial virus infection in adults and the elderly in the United Kingdom. *BMC Infect Dis*. 2015;15:443.

6. Matias G, Taylor R, Haguinet F, Schuck-Paim C, Lustig R, Shinde V. Estimates of hospitalization attributable to influenza and RSV in the US during 1997-2009, by age and risk status. *BMC Public Health*. 2017;17(1):271.
